# Supplementary material for: DREAM represses distinct targets by cooperating with different THAP domain proteins
Source: Cell Rep. 2021 Oct 19;37(3):109835. doi: 10.1016/j.celrep.2021.109835 (PMC8552245; doi:10.1016/j.celrep.2021.109835)
Supplement: Document S1. Figures S1–S5 [file mmc1.pdf]

Cell Reports, Volume *37*

Supplemental information

DREAM represses distinct targets

by cooperating with different THAP domain proteins

Csenge Gal, Francesco Nicola Carelli, Alex Appert, Chiara Cerrato, Ni Huang, Yan Dong, Jane Murphy, Andrea Frapporti, and Julie Ahringer

Figure S1

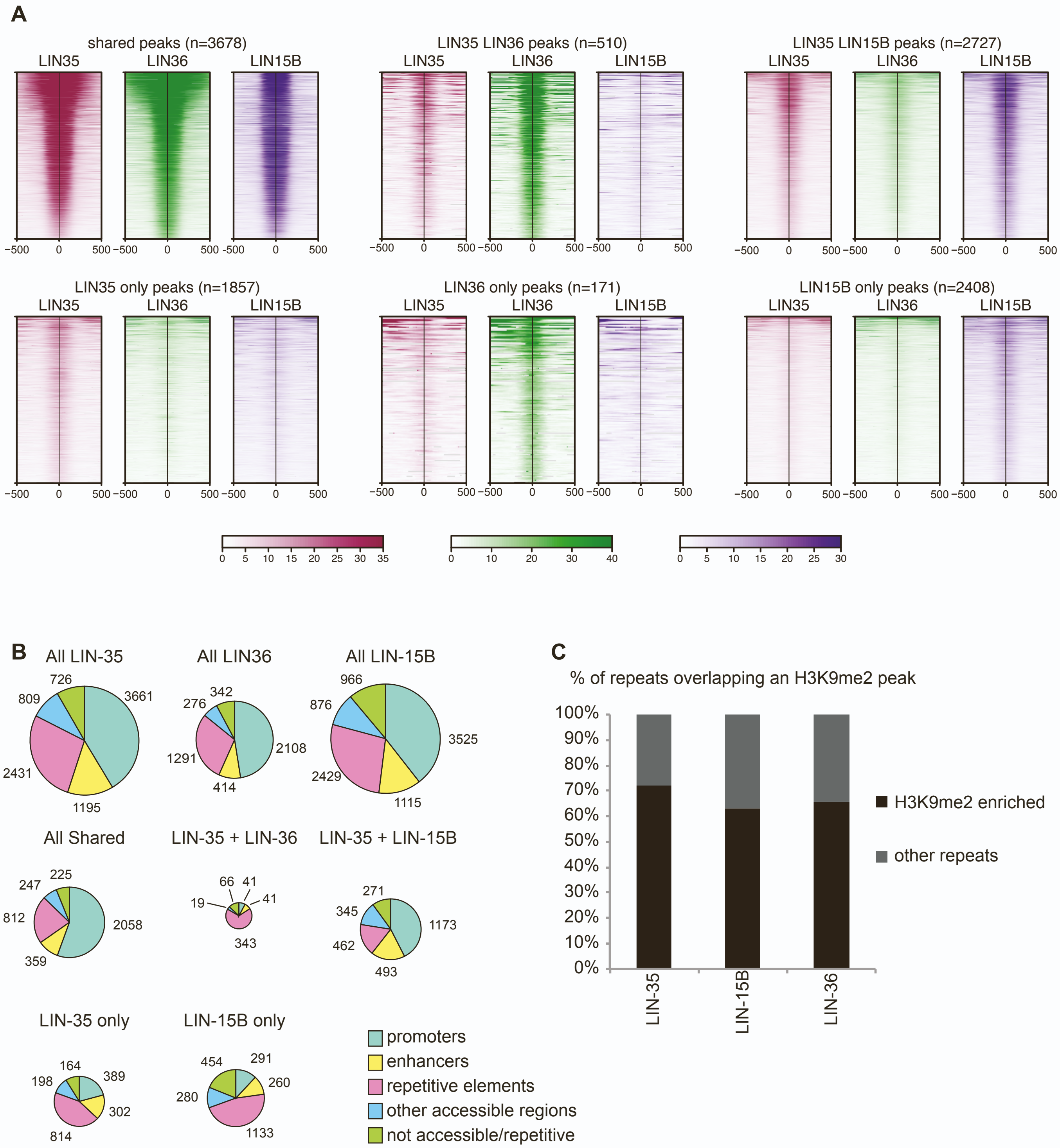

A
shared peaks (n=3678)
LIN35 LIN36 peaks (n=510)
LIN35 LIN15B peaks (n=2727)
LIN35 only peaks (n=1857)
LIN36 only peaks (n=171)
LIN15B only peaks (n=2408)
LIN35
LIN36
LIN15B
-500 0 500
B
All LIN-35
All LIN36
All LIN-15B
All Shared
LIN-35 + LIN-36
LIN-35 + LIN-15B
LIN-35 only
LIN-15B only
promoters
enhancers
repetitive elements
other accessible regions
not accessible/repetitive
C
% of repeats overlapping an H3K9me2 peak
H3K9me2 enriched
other repeats
LIN-35
LIN-15B
LIN-36

Figure S1. LIN-35, LIN-36 and LIN-15B co-localize extensively on chromatin, related to Figure 1. (A) Heatmaps of BEADS normalized ChIP-seq coverage centred over the indicated regions. Tracks are from combined replicates. We note that signal at single factor sites is generally weak and therefore confidence that other factors are not present is not high. (B) Assignments of peaks to features in the genome. Peaks were first overlapped with regulatory elements identified in Janes *et al*. 2018, then with repetitive elements from Dfam2.0. (C) Fraction of LIN-35, LIN-15B and LIN-36-bound repeats (from Dfam 2.0) overlapping H3K9me2 ChIP-seq peaks.

Figure S2

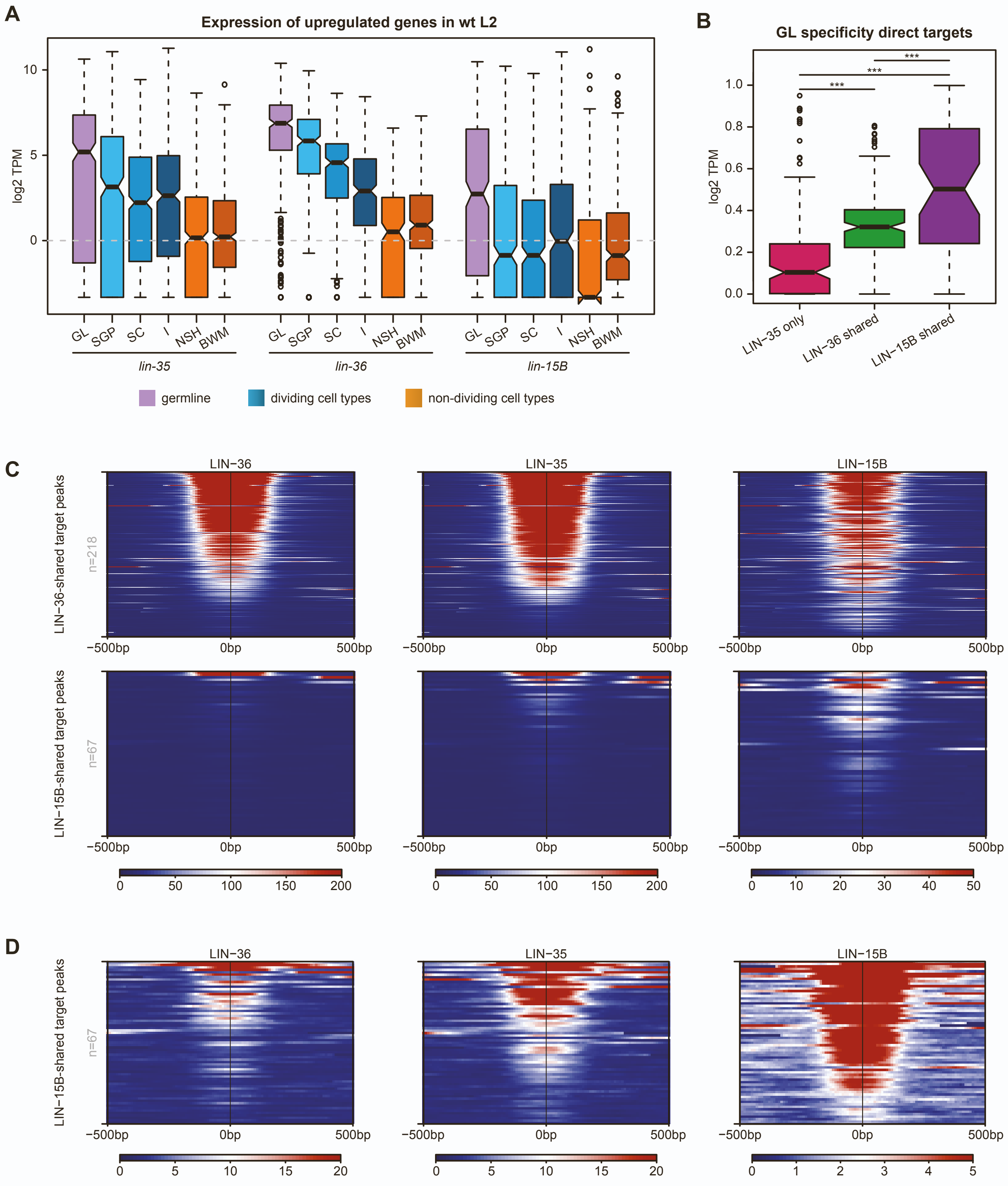

A
Expression of upregulated genes in wt L2
log2 TPM
10
5
0
GL SGP SC I NSH BWM
lin-35
lin-36
lin-15B
germline
dividing cell types
non-dividing cell types
B
GL specificity direct targets
log2 TPM
1.0
0.8
0.6
0.4
0.2
0.0
***
***
***
LIN−35 only
LIN−36 shared
LIN−15B shared
C
LIN−36
LIN−35
LIN−15B
LIN-36-shared target peaks
n=218
LIN−15B-shared target peaks
n=67
−500bp
0bp
500bp
0 50 100 150 200
0 10 20 30 40 50
D
LIN−36
LIN−35
LIN−15B
LIN−15B-shared target peaks
n=67
0 5 10 15 20
0 1 2 3 4 5

Figure S2. Expression and binding profile of LIN-35, LIN-36 and LIN-15B targets, related to Figure 1. (A) Expression level (measured as log2 TPM) of genes upregulated in *lin-35*, *lin-36(we36)*, and *lin-15B* in the germline and in different types of dividing (SGP: somatic gonad precursors, SC: seam cells, I: intestine) and non-dividing (NSH: non-seam hypodermis, BWM: body wall muscle) cell types. The dashed grey line indicates a TPM value of 1. Expression difference between germline and any other tissue was significant for LIN-35, LIN-36 and LIN-15B targets (Benjamini-Hochberg adjusted Mann-Whitney test $P<10^{-3}$) (B) Germline expression specificity (calculated as expression in germline / sum of expression in any cell type) of LIN-35-specific, LIN-36-shared and LIN-15B-shared direct targets. (C) and (D) Heatmaps of BEADS normalized ChIP-seq coverage centred over the LIN-35+LIN-36 and LIN35+LIN-15B peaks associated to the promoters of LIN-36-shared (C) and LIN-15B-shared (C and D, in different scales) direct targets. Significant differences (Wilcoxon rank sum test): ***: $P<0.001$. Expression data for panels (A, B) are from Cao et al, 2017.

Figure S3

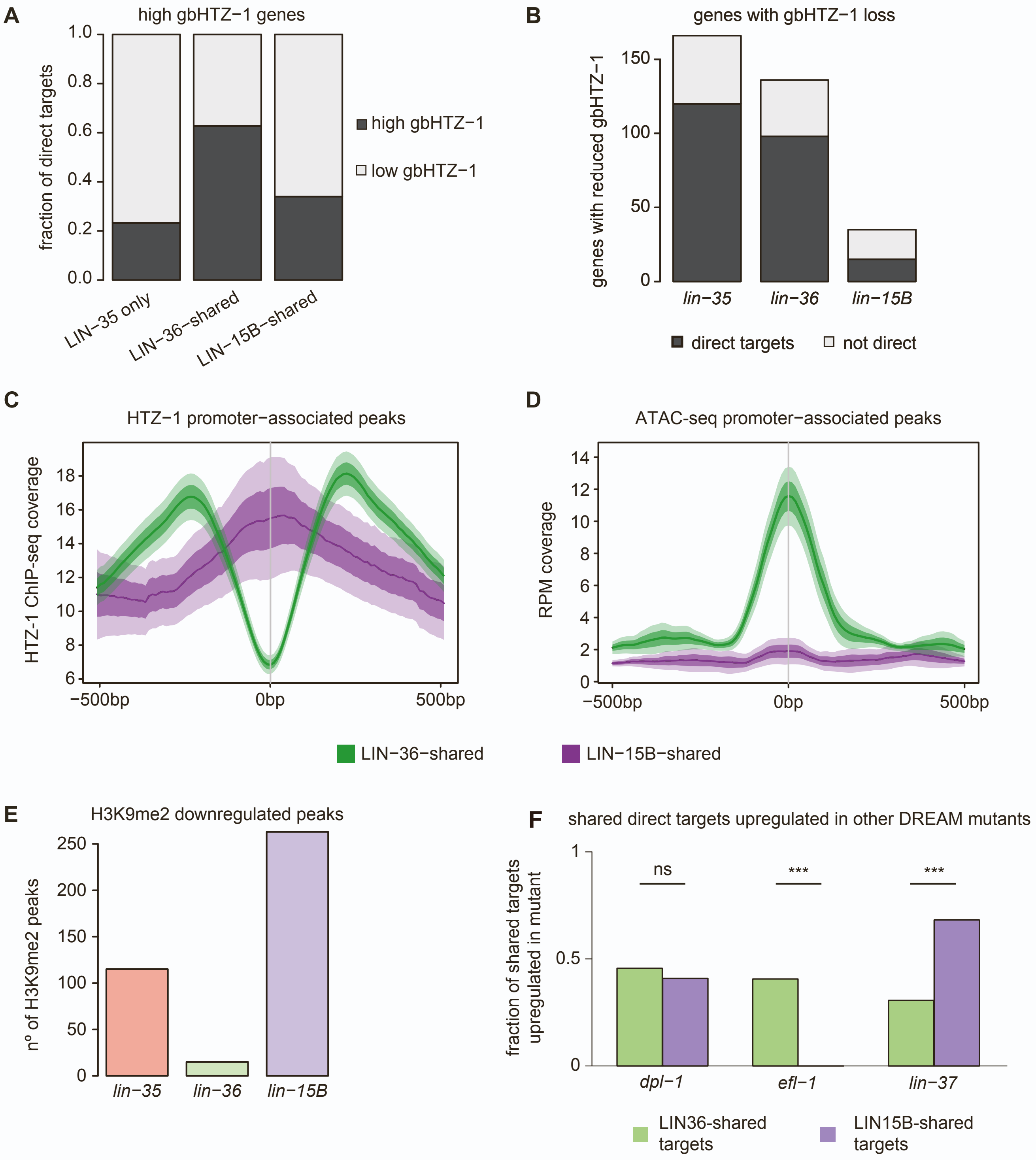
A
high gbHTZ−1 genes
fraction of direct targets
1.0
0.8
0.6
0.4
0.2
0.0
high gbHTZ−1
low gbHTZ−1
LIN−35 only
LIN−36−shared
LIN−15B−shared
B
genes with gbHTZ−1 loss
genes with reduced gbHTZ−1
150
100
50
0
lin−35
lin−36
lin−15B
direct targets
not direct
C
HTZ−1 promoter−associated peaks
HTZ-1 ChIP-seq coverage
18
16
14
12
10
8
6
−500bp
0bp
500bp
D
ATAC-seq promoter−associated peaks
RPM coverage
14
12
10
8
6
4
2
0
−500bp
0bp
500bp
LIN−36−shared
LIN−15B−shared
E
H3K9me2 downregulated peaks
nº of H3K9me2 peaks
250
200
150
100
50
0
lin−35
lin−36
lin−15B
F
shared direct targets upregulated in other DREAM mutants
fraction of shared targets upregulated in mutant
1
0.5
0
ns
***
***
dpl−1
efl−1
lin−37
LIN36-shared targets
LIN15B-shared targets

Figure S3. Different silencing mechanisms of LIN-36-shared targets and LIN-15B-shared targets, related to Figure 2. (A) fraction of LIN-35-specific, LIN-36-shared and LIN-15B-shared direct targets with high (top 10% across all coding genes, dark grey) or low (bottom 90%, light grey) levels of gbHTZ-1. (B) Number of coding genes showing a significant reduction in gbHTZ-1 levels in the respective mutants. Dark grey bars indicate direct targets. (C) Signal plot of BEADS normalised HTZ-1 ChIP-seq coverage calculated over the LIN-35+LIN-36 and LIN35+LIN-15B peaks associated to the promoters of LIN-36-shared (green) and LIN-15B-shared (purple) direct targets. (D) Signal plot of ATAC-seq signal (in RPM coverage) from L1-staged larvae over the LIN-35+LIN-36 and LIN35+LIN-15B peaks associated to the promoters of LIN-36-shared (green) and LIN-15B-shared (purple) direct targets. ATAC-seq data from Janes et al., 2018. (E) Number of LIN-35, LIN-36 and/or LIN-15B peaks showing a significant reduction in H3K9me2 levels in mutants. (F) Fraction of LIN-36-shared (green) and LIN-15B-shared (purple) direct targets showing upregulated expression in *dpl-1*, *efl-1* and *lin-37* mutants. Significant differences (LIN-36-shared vs LIN-15B-shared fraction, Fisher's exact test with Benjamini-Hochberg correction): ***: $P<0.001$; ns: $P>0.05$.

Figure S4

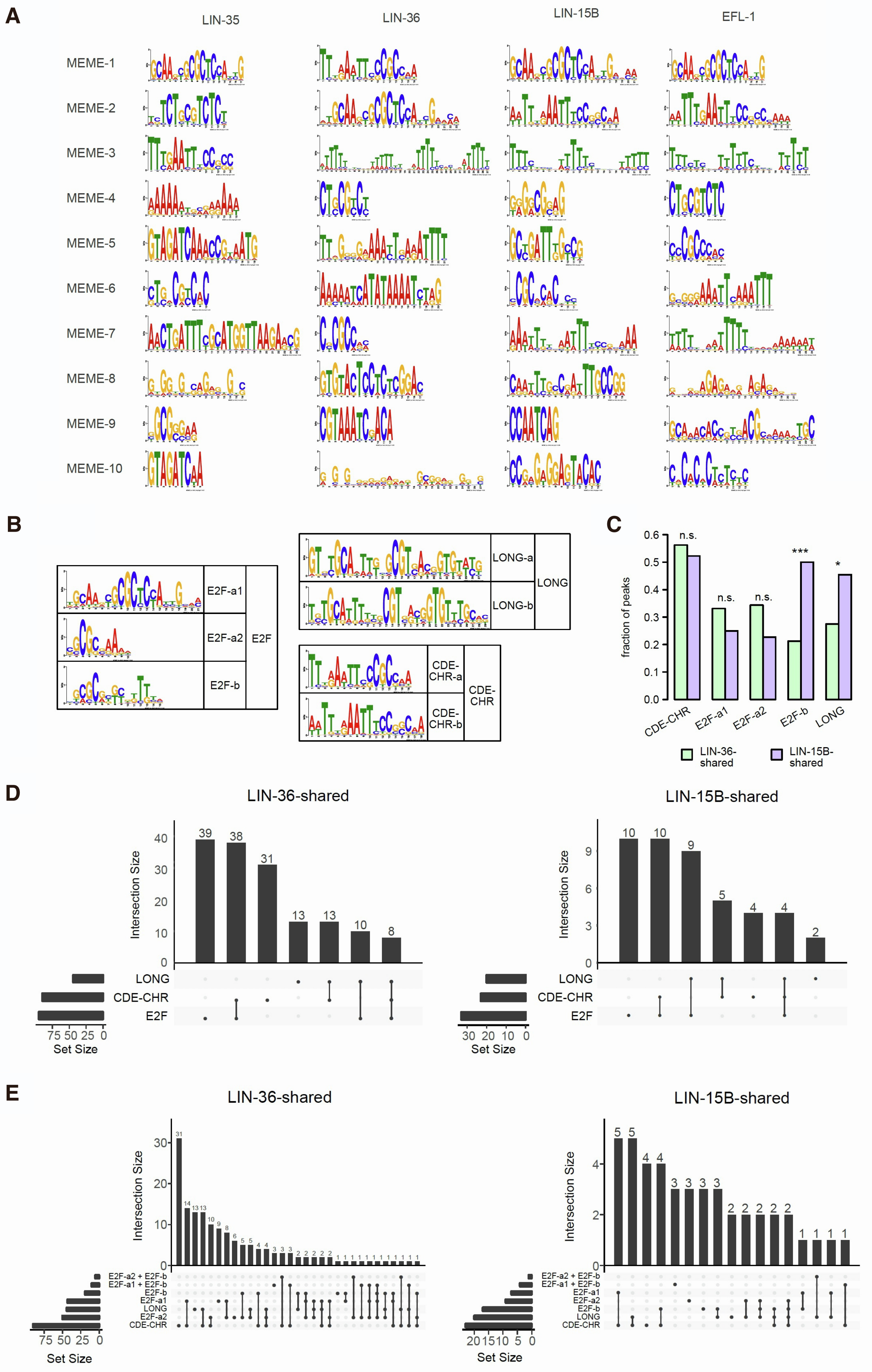

A
LIN-35
LIN-36
LIN-15B
EFL-1
MEME-1
MEME-2
MEME-3
MEME-4
MEME-5
MEME-6
MEME-7
MEME-8
MEME-9
MEME-10
B
E2F-a1
E2F-a2
E2F-b
E2F
LONG-a
LONG-b
LONG
CDE-CHR-a
CDE-CHR-b
CDE-CHR
C
fraction of peaks
n.s.
n.s.
n.s.
***
*
CDE-CHR
E2F-a1
E2F-a2
E2F-b
LONG
LIN-36-shared
LIN-15B-shared
D
LIN-36-shared
Intersection Size
39
38
31
13
13
10
8
LONG
CDE-CHR
E2F
Set Size
LIN-15B-shared
10
10
9
5
4
4
2
E
LIN-36-shared
LIN-15B-shared
E2F-a2 + E2F-b
E2F-a1 + E2F-b
E2F-b
E2F-a1
LONG
E2F-a2
CDE-CHR

Figure S4, Motifs enriched at LIN-35, LIN-36, LIN-15B and EFL-1 peaks, related to Figure 1. (A) Logos of motifs enriched in full sets of LIN-35, LIN-36, LIN-15B and EFL-1 peaks. (B) E2F, LONG and CDE-CHR motif variants enriched at LIN-36-shared and LIN-15B-shared target promoters. (C) Fraction of LIN-36-shared and LIN-15B-shared promoter-associated peaks containing each motif. For CDE-CHR and LONG motifs, we included sites corresponding to both variant. Significant differences (Fisher's exact test with Benjamini-Hochberg correction): ***: $P < 0.001$; *: $P < 0.05$; ns: $P > 0.05$. (D-E) UpSet plots showing the co-occurrence of each motif class (D) or of individual E2F variants with LONG and CDE-CHR motifs (E) in LIN-36-shared and LIN-15B-shared target peaks.

Figure S5

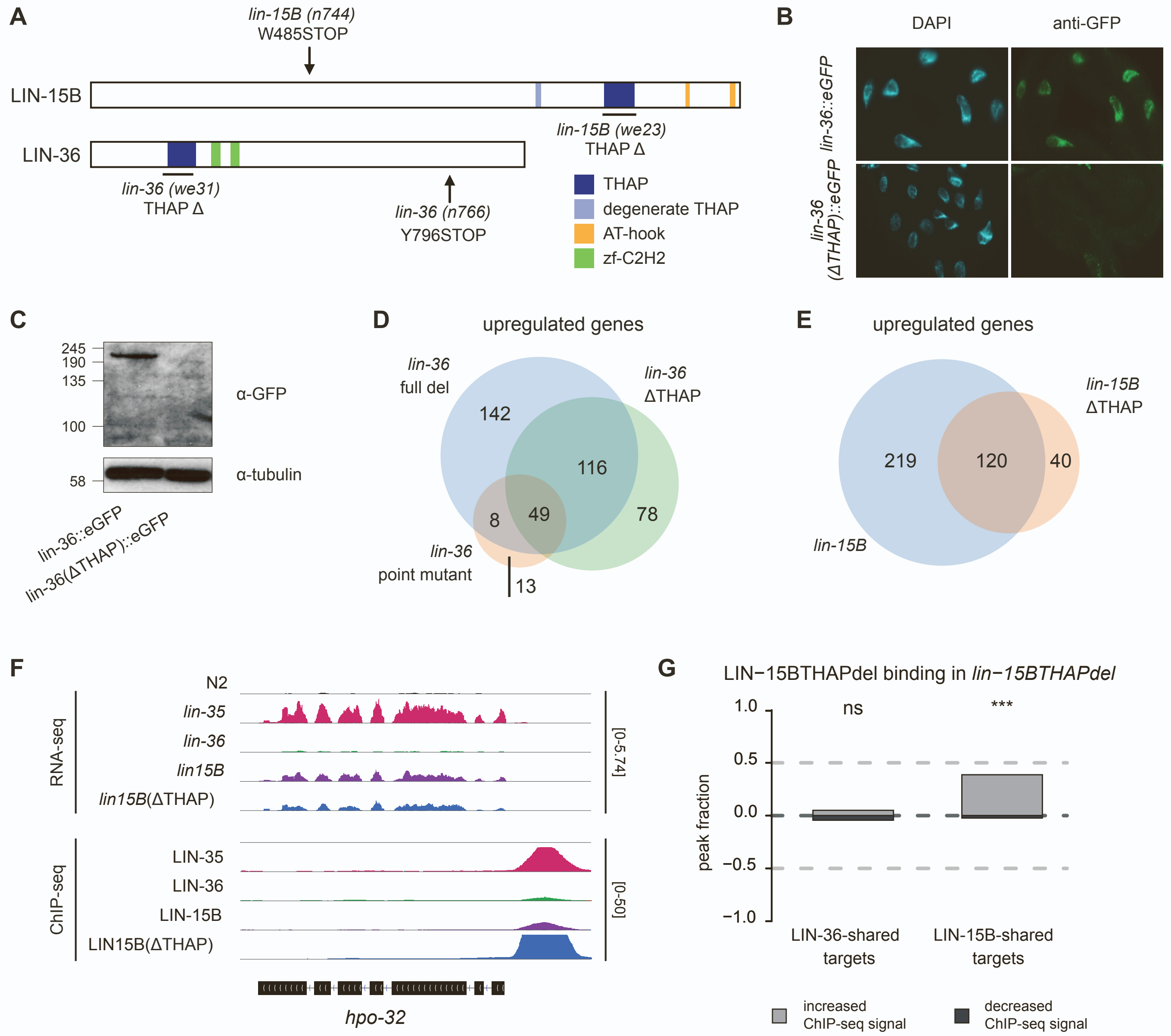

A
lin-15B (n744)
W485STOP
LIN-15B
lin-15B (we23)
THAP Δ
LIN-36
lin-36 (we31)
THAP Δ
lin-36 (n766)
Y796STOP
THAP
degenerate THAP
AT-hook
zf-C2H2
B
DAPI
anti-GFP
lin-36::eGFP
lin-36 (ΔTHAP)::eGFP
C
245
190
135
100
58
α-GFP
α-tubulin
lin-36::eGFP
lin-36(ΔTHAP)::eGFP
D
upregulated genes
lin-36 full del
lin-36 ΔTHAP
lin-36 point mutant
142
116
8
49
78
13
E
upregulated genes
lin-15B ΔTHAP
lin-15B
219
120
40
F
RNA-seq
N2
lin-35
lin-36
lin15B
lin15B(ΔTHAP)
[0-5.74]
ChIP-seq
LIN-35
LIN-36
LIN-15B
LIN15B(ΔTHAP)
[0-50]
hpo-32
G
LIN−15BTHAPdel binding in lin−15BTHAPdel
1.0
0.5
0.0
−0.5
−1.0
peak fraction
ns
***
LIN-36-shared targets
LIN-15B-shared targets
increased ChIP-seq signal
decreased ChIP-seq signal

Figure S5. Effects of THAP domain deletion in LIN-36 and LIN-15B, related to Figure 3. (A) Diagram of the LIN-15B and LIN-36 proteins, illustrating their predicted DNA binding domains and the deletions generated in this study. Arrows indicate the positions of the premature stop codons in the corresponding alleles. The lin-15B(ΔTHAP) allele did not remove the highly degenerate, upstream THAP-like domain. (B) Anti-GFP immunofluorescence of the LIN-36::eGFP fusion protein in the *lin-36::eGFP* and *lin-36(ΔTHAP)::eGFP* strains. No GFP signal was detected in *lin-36(ΔTHAP)::eGFP* worms. C) Western blot with anti-GFP antibody was used to detect the LIN-36::eGFP and LIN-36(ΔTHAP)::eGFP proteins; the band corresponding to the LIN-36(ΔTHAP)::eGFP protein was not detected. (D) and (E) Overlap between genes upregulated in (D) *lin-36* or (E) *lin-15B* mutant strains. (F) IGV view of a representative LIN-15B-shared direct target. Factor-specific ChIP-seq enrichment shown as BEADS-normalized coverage. RNA sequencing data depict read-depth normalized coverage from combined replicates. (G) Fraction of LIN-36-shared (left) and LIN-15B-shared (right) promoter-associated LIN15B(ΔTHAP) peaks showing a significant difference in occupancy in *lin-15B(ΔTHAP)* mutants. Significant differences (up- vs downregulated fraction, Fisher's exact test with Benjamini-Hochberg correction): ***: $P < 0.001$; ns: $P > 0.05$.
