# Supplementary material for: DREAM represses distinct targets by cooperating with different THAP domain proteins
Source: Cell Rep. 2021 Oct 19;37(3):109835. doi: 10.1016/j.celrep.2021.109835 (PMC8552245; doi:10.1016/j.celrep.2021.109835)
Supplement: Document S2. Article plus supplemental information [file mmc8.pdf]

Cell Reports

Report

DREAM represses distinct targets by cooperating with different THAP domain proteins

Graphical abstract

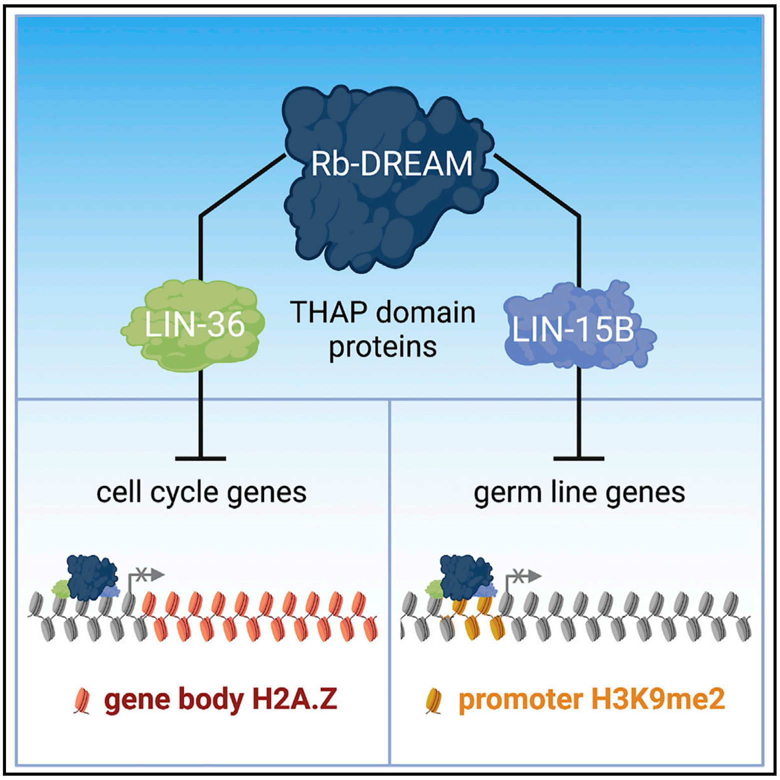

Highlights

- THAP domain proteins LIN-36 and LIN-15B cooperate with the Rb-DREAM complex
- LIN-36 and LIN-15B repress distinct sets of DREAM targets via different mechanisms
- With LIN-36, DREAM represses cell-cycle genes through gene body enrichment of H2A.Z
- With LIN-15B, DREAM represses germline genes through H3K9me2 promoter marking

Authors

Csenge Gal, Francesco Nicola Carelli, Alex Appert, ..., Jane Murphy, Andrea Frapporti, Julie Ahringer

Correspondence

ja219@cam.ac.uk

In brief

Gal et al. show that two THAP domain proteins are key mediators of retinoblastoma-DREAM function in *C. elegans*, repressing distinct targets by different mechanisms. With LIN-36, DREAM represses cell-cycle genes through gene body enrichment of H2A.Z; with LIN-15B, DREAM represses germline-specific genes in the soma through H3K9me2 promoter marking.

Gal et al., 2021, Cell Reports *37*, 109835
October 19, 2021 © 2021 The Author(s).
https://doi.org/10.1016/j.celrep.2021.109835

CellPress

Cell Reports

CellPress
OPEN ACCESS

Report

DREAM represses distinct targets by cooperating with different THAP domain proteins

Csenge Gal,[1,2] Francesco Nicola Carelli,[1,2] Alex Appert,[1] Chiara Cerrato,[1] Ni Huang,[1] Yan Dong,[1] Jane Murphy,[1] Andrea Frapporti,[1] and Julie Ahringer[1,3,*]
[1]Wellcome Trust/Cancer Research UK Gurdon Institute and Department of Genetics, University of Cambridge, Cambridge, UK
[2]These authors contributed equally
[3]Lead contact
*Correspondence: ja219@cam.ac.uk
https://doi.org/10.1016/j.celrep.2021.109835

SUMMARY

The DREAM (dimerization partner [DP], retinoblastoma [Rb]-like, E2F, and MuvB) complex controls cellular quiescence by repressing cell-cycle and other genes, but its mechanism of action is unclear. Here, we demonstrate that two *C. elegans* THAP domain proteins, LIN-15B and LIN-36, co-localize with DREAM and function by different mechanisms for repression of distinct sets of targets. LIN-36 represses classical cell-cycle targets by promoting DREAM binding and gene body enrichment of H2A.Z, and we find that DREAM subunit EFL-1/E2F is specific for LIN-36 targets. In contrast, LIN-15B represses germline-specific targets in the soma by facilitating H3K9me2 promoter marking. We further find that LIN-36 and LIN-15B differently regulate DREAM binding. In humans, THAP proteins have been implicated in cell-cycle regulation by poorly understood mechanisms. We propose that THAP domain proteins are key mediators of Rb/DREAM function.

INTRODUCTION

During animal development, cell proliferation is tightly controlled, and differentiated cells spend the majority of the time in a quiescent, nondividing state. The regulation of quiescence is crucial, as uncontrolled proliferation can lead to tumor formation, while premature senescence is associated with aging. Despite its importance, mechanisms of quiescence regulation remain poorly understood.

The retinoblastoma (Rb) family of pocket proteins (Rb, p130, and p107) are key regulators of the cell-division cycle, regulating progression from G_1 to S phase and maintaining the G_0 state via transcriptional repression of proliferation-promoting genes (Dick and Rubin, 2013). The majority of cancers disable Rb protein function or alter its regulation (Liu et al., 2013; Nor Rashid et al., 2011; Sadasivam and DeCaprio, 2013). Loss of Rb also leads to developmental defects (Du et al., 1996; Lee et al., 1992; Lu and Horvitz, 1998). A mechanistic understanding of Rb proteins is essential for understanding their roles in normal development and cancerous transformations.

Of the Rb family of proteins p130 is the most highly expressed during stable cell-cycle arrest, such as quiescence and senescence, through which it represses proliferation-promoting genes as part of a repressive complex called DREAM (dimerization partner [DP], Rb-like, E2F, and MuvB; Lewis et al., 2004; Litovchick et al., 2007, 2011; Schmit et al., 2007). In different organisms, disruption of DREAM leads to developmental defects, an increase in genomic instability, tumorigenesis, and lethality (Hauser et al., 2012; Malumbres and Barbacid, 2009; Reichert et al., 2010; Schade et al., 2019). The mechanisms by which DREAM functions in these different processes is unclear.

The DREAM complex is highly conserved in subunit composition and function in animals (Sadasivam and DeCaprio, 2013). Mammalian DREAM is composed of the Rb-like protein p130 (or p107 in the absence of p130), an E2F (E2F4/E2F5), a DP protein, and MuvB proteins (LIN9, LIN54, LIN52, LIN37, and RBBP4) (Litovchick et al., 2007; Schmit et al., 2007). As in mammals, *C. elegans* DREAM (LIN-35/Rb, DPL-1/DP, EFL-1/E2F, LIN-9, LIN-37, LIN-53, LIN-54, and LIN-52) represses cell-cycle-specific genes and others, including germline genes in somatic tissues (Goetsch et al., 2017; Korenjak et al., 2004; Latorre et al., 2015; Rechtsteiner et al., 2019). Since DREAM itself contains no known enzymatic activity, it is thought to repress targets through effector proteins. Indeed, such a role has been proposed for the Sin3B-HDAC complex in mammalian cells (Bainor et al., 2018; Rayman et al., 2002). In addition, we previously showed that repression of a subset of *C. elegans* DREAM targets involves deposition of HTZ-1/H2A.Z on their gene bodies (Latorre et al., 2015). To further mechanistic understanding, we undertook an RNAi screen for additional factors needed for repression of a DREAM target. Here, we show that two THAP domain proteins function with DREAM by different mechanisms to repress distinct sets of targets.

RESULTS

An RNAi screen identifies novel regulators of Rb/DREAM targets

To identify proteins involved in DREAM transcriptional repression, we constructed a DREAM-regulated reporter gene by fusing the promoter of the target *sep-1* to a histone-EGFP coding region and then carried out an RNAi screen for genes

This is an open access article under the CC BY license (http://creativecommons.org/licenses/by/4.0/).

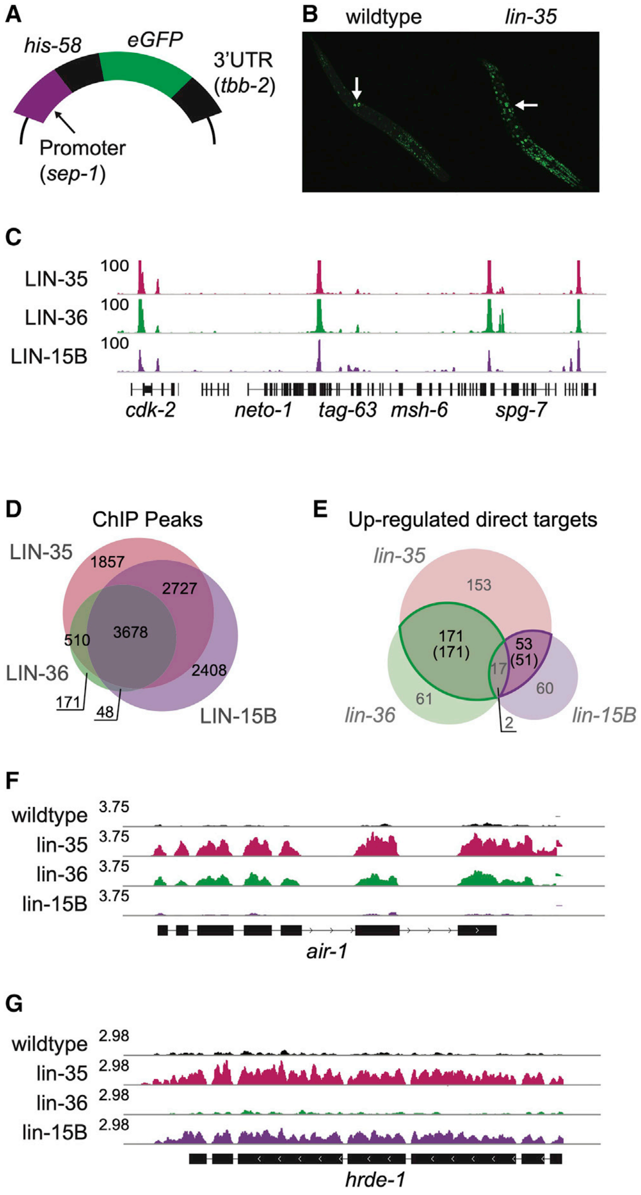

Figure 1. THAP-domain proteins LIN-36 and LIN-15B regulate Rb/DREAM targets

(A) *p-sep-1::eGFP* DREAM target reporter gene used for the RNAi screen.

(B) *lin-35* mutant animals have increased expression the *p-sep-1::eGFP* reporter relative to wild type. Arrows indicate the two germ cells in starved L1 animals.

(C) IGV view of linear BEADS-normalized ChIP-seq coverage for the indicated factors.

(D) Overlap of ChIP-seq peaks called for the indicated factors.

(E) Overlap between direct targets in the indicated mutants. Numbers in parentheses indicate LIN-36-shared targets (green) and LIN-15B-shared targets (purple) (see STAR Methods).

(F and G) IGV view of RNA sequencing (RNA-seq) data (in RPM, reads per million mapped reads) of a LIN-36-shared (F) and LIN-15B-shared (G) target.

needed for reporter repression (Figure 1A). The screen was carried out in quiescent starved L1 larvae, which contain 550 nondividing somatic cells and 2 germ cells. In wild-type starved L1s, the *P-sep-1::his-58::eGFP* transgene is expressed in the germline and largely repressed in the soma (Figure 1B). In *lin-35/Rb* mutants, reporter expression is increased in the soma compared to the wild type (Figure 1B). The RNAi screen targeted 1,104 genes encoding nuclear proteins to identify genes that are required to prevent somatic expression of the *P-sep-1::his-58::eGFP* reporter (see STAR Methods). Following RNAi knockdown, EGFP expression was measured using a worm sorter, which identified 36 genes for which knockdown caused reporter de-repression (Table S1), including seven out of eight DREAM components (*lin-35*/Rb, *efl-1*, *dpl-1*, *lin-54*, *lin-9*, *lin-37*, and *lin-53*), validating the screen. Others include components of the MCM complex, a number of RNA-binding proteins, proteins required for kinetochore function, and *lin-36*, which encodes a THAP-domain-containing protein.

LIN-36 was of particular interest, as its loss has been shown to cause cell-cycle defects similar to those of DREAM mutants (Boxem and van den Heuvel, 2002), but it has not been well characterized. LIN-36 contains a THAP domain, which is an atypical zinc-finger DNA-binding domain derived from a transposase (Clouaire et al., 2005; Roussigne et al., 2003). *C. elegans* has 17 THAP- or THAP-like-domain-containing proteins, of which 7 have been shown to genetically interact with *lin-35*/Rb (Table S1) (Boxem and van den Heuvel, 2002; Ceron et al., 2007; Chesney et al., 2006; Ouellet and Roy, 2007; Poulin et al., 2005; Reddy and Villeneuve, 2004; Saito et al., 2004), suggesting a broad relationship between THAP domain proteins and LIN-35/Rb. Humans have 12 THAP domain proteins, THAP0 to THAP11, which have been implicated in diverse cellular processes, including the regulation of cell-cycle genes (Cayrol et al., 2007; Ceron et al., 2007). Disruption of THAP proteins has also been linked to various diseases, including cancers (Balakrishnan et al., 2009; Gervais et al., 2013; Richter et al., 2017). We used RNAi to test whether other THAP domain genes are required for repression of the *P-sep-1::his-58::eGFP* reporter and found that LIN-15B is also needed (Table S1). We note that GON-14 protein shares significant similarity with LIN-15B but lacks key conserved residues in its degenerate THAP domain (Chesney et al., 2006; Clouaire et al., 2005), and RNAi of *gon-14* did not increase reporter expression. Previous work showed that LIN-15B and LIN-35 share some transcriptional targets (Rechtsteiner et al., 2019), and LIN-15B has been implicated in negative regulation of the G_1/S transition of the cell cycle (Boxem and van den Heuvel, 2002). Here, we investigate the roles of LIN-36 and LIN-15B in the repression of DREAM targets.

LIN-36 and LIN-15B co-localize with LIN-35

To explore the relationships among LIN-35, LIN-36, and LIN-15B, we first compared their genome-wide binding patterns using chromatin immunoprecipitation sequencing (ChIP-seq) in wild-type starved L1 animals using antibodies to LIN-35 and LIN-15B and detecting LIN-36 by an endogenous GFP-tag (see STAR Methods). We found that LIN-36 and LIN-15B both show a high degree of overlap with LIN-35, with 95% of LIN-36 and 72% of LIN-15B peaks overlapping a LIN-35 peak (Figures 1C, 1D, and S1A; Table S2). For each factor, most (59%–69%) peaks overlap

a promoter or enhancer, with much of the remainder localizing to repetitive elements (Figure S1B). Many of the repeat regions are marked by H3K9me2, supporting a possible connection between H3K9me2 and DREAM (Figure S1C; Rechtsteiner et al., 2019).

LIN-36 and LIN-15B repress discrete sets of LIN-35 targets

We next compared the effects of loss of LIN-35, LIN-36, and LIN-15B on gene expression (Table S3). We used available null alleles *lin-35(n745)* and *lin-15B(n744)* and generated full-deletion allele *lin-36(we36)* using CRISPR-Cas9 gene editing (see STAR Methods). We also profiled the partial loss-of-function allele *lin-36(n766)*. For all mutants, we observed that the primary effect was loss of repression (Table S3) and hence focused our work on direct repressed targets, which are defined as genes upregulated in *lin-35*, *lin-36*, or *lin-15B* mutants and bound by the corresponding factor (see STAR Methods).

We observed that repressed targets of LIN-36 or LIN-15B each significantly overlap LIN-35/Rb targets (>21-fold enrichment, hypergeometric test $p < 10^{-76}$), but strikingly, genes regulated by LIN-36 and LIN-15B are mostly distinct (Figures 1E and 1F). Here, we focus on genes directly regulated by LIN-35 and LIN-36 (LIN-36-shared targets; n = 171) or LIN-35 and LIN-15B (LIN-15B-shared targets; n = 51) (Table S3). Using gene ontology (GO) analyses, we found that LIN-36-shared targets are highly enriched for cell-cycle and cell-division terms (Table S3). No enriched GO terms were found for LIN-15B-shared targets (Table S3); however, we observed that they have high germline expression specificity (Figures S2A and S2B; Table S3). LIN-36-shared targets and LIN-15B-shared targets also dramatically differ in the binding profiles of LIN-35, LIN-36, and LIN-15B, with higher signal for all three factors at LIN-36-shared targets compared to LIN-15B-shared targets (Figures S2C and S2D). Altogether, these observations suggest that LIN-15B-shared and LIN-36-shared genes represent two distinct classes of DREAM targets with potentially different regulation and functional roles.

LIN-36 maintains gene body HTZ-1

We previously showed that transcriptional repression of a subset of DREAM target genes involves LIN-35-dependent enrichment of the histone variant H2A.Z/HTZ-1 over their gene bodies (gbHTZ-1) (Latorre et al., 2015). To assess whether LIN-36 and/or LIN-15B act with LIN-35 in facilitating gbHTZ-1, we first asked whether gene body enrichment of HTZ-1 was associated with either set of shared targets. Indeed, we observed that LIN-36-shared targets were more enriched for high gbHTZ-1 than LIN-15B-shared targets (Figures 2A and S3A; Table S4).

Evaluating gbHTZ-1 levels on targets in wild-type and mutant starved L1s, we found that the majority of LIN-36-shared targets require both LIN-35 and LIN-36 for high gbHTZ-1 levels, but loss of LIN-15B had no obvious effect at these loci (Figures 2B, 2C, and S3B; Table S4). In contrast, although some LIN-15B-shared targets required LIN-35 and LIN-15B for gbHTZ-1, these were in the minority (Figures 2B, D). Overall, approximately half (144/293) of all DREAM targets characterized by high gbHTZ-1 correspond to LIN-36-shared targets, and both LIN-36 and LIN-35 function to facilitate the recruitment or maintenance of HTZ-1 over these targets.

LIN-15B promotes H3K9me2 marking for repression of its targets

In addition to differences in gbHTZ-1, we observed a substantial difference in the HTZ-1 profiles over the promoters of different sets of DREAM targets. While LIN-36-shared targets have a bimodal distribution of HTZ-1 flanking the associated LIN-35 and LIN-36 peaks in wild-type animals, HTZ-1 was instead centrally enriched at LIN-15B-shared target peaks (Figure S3C). The HTZ-1 profiles suggest that promoters of LIN-36-shared and LIN-15B-shared targets have different chromatin states. Indeed, whereas LIN-36-shared target peaks showed high DNA accessibility, peaks associated with promoters of LIN-15B-shared targets had low DNA accessibility, indicative of a generally closed chromatin conformation (Figure S3D).

We considered that repression of LIN-15B-shared targets could involve a chromatin-based repression mechanism involving H3K9me2, as previous work showed that LIN-15B facilitates H3K9me2 marking of some DREAM target promoters, although the relevance of H3K9me2 at these genes was not determined (Rechtsteiner et al., 2019). In addition, we observed that LIN-35, LIN-36, and LIN-15B associate with H3K9me2-marked repeats (Figure S1C).

Investigating this connection, we found that H3K9me2 was strongly enriched at LIN-15B-shared, but not LIN-36-shared, target promoters (Figure 2F; Table S4). We further found that H3K9me2 marking at LIN-15B-shared target promoters is dependent on LIN-15B (Figure 2G). Notably, H3K9me2 was significantly reduced at 50% of LIN-15B-shared target promoters in *lin-15B* mutants, and to a lower extent in *lin-35* mutants (Figure 2G), whereas little effect was seen in *lin-36* mutants or at LIN-36-shared targets.

To test the functional relevance of H3K9me2 in target repression, we profiled gene expression in mutants of *met-2*, which encodes the major H3K9me2 histone methyltransferase (Bessler et al., 2010). We found that LIN-15B-shared targets had higher expression in *met-2* mutants, with 43% being significantly upregulated, whereas *met-2* loss had little effect on LIN-36-shared targets (Figure 2H; Table S3). Mechanistically, these results implicate LIN-15B and DREAM in directing repression of their shared targets via MET-2-dependent H3K9me2 promoter marking.

EFL-1/E2F function is specific for LIN-36-shared targets

We next investigated whether repression of LIN-36-shared and LIN-15B-shared targets differed in their requirement for DREAM components. The DREAM complex consists of DNA-binding protein EFL-1/E2F and partner DPL-1/DP1, which are proposed to be bridged to the MuvB sub-complex (LIN-9, LIN-37, LIN-53, LIN-54, and LIN-52) by LIN-35/Rb (Goetsch et al., 2017). To evaluate requirements for different components, we compared gene expression changes among mutants of *lin-35/Rb*, *efl-1*, *dpl-1*, and MuvB sub-complex component *lin-37* (Table S3). We found that changes in *dpl-1* and *lin-37* mutants were similar to those of *lin-35* mutants, suggesting a common mechanism. Both LIN-36-shared and LIN-15B-shared targets were derepressed in the two mutants, suggesting that DPL-1 and LIN-37 participate in LIN-35 core roles (Figure S3F). In stark contrast, *efl-1* mutants only derepressed LIN-36-shared targets (Figure S3F). The striking

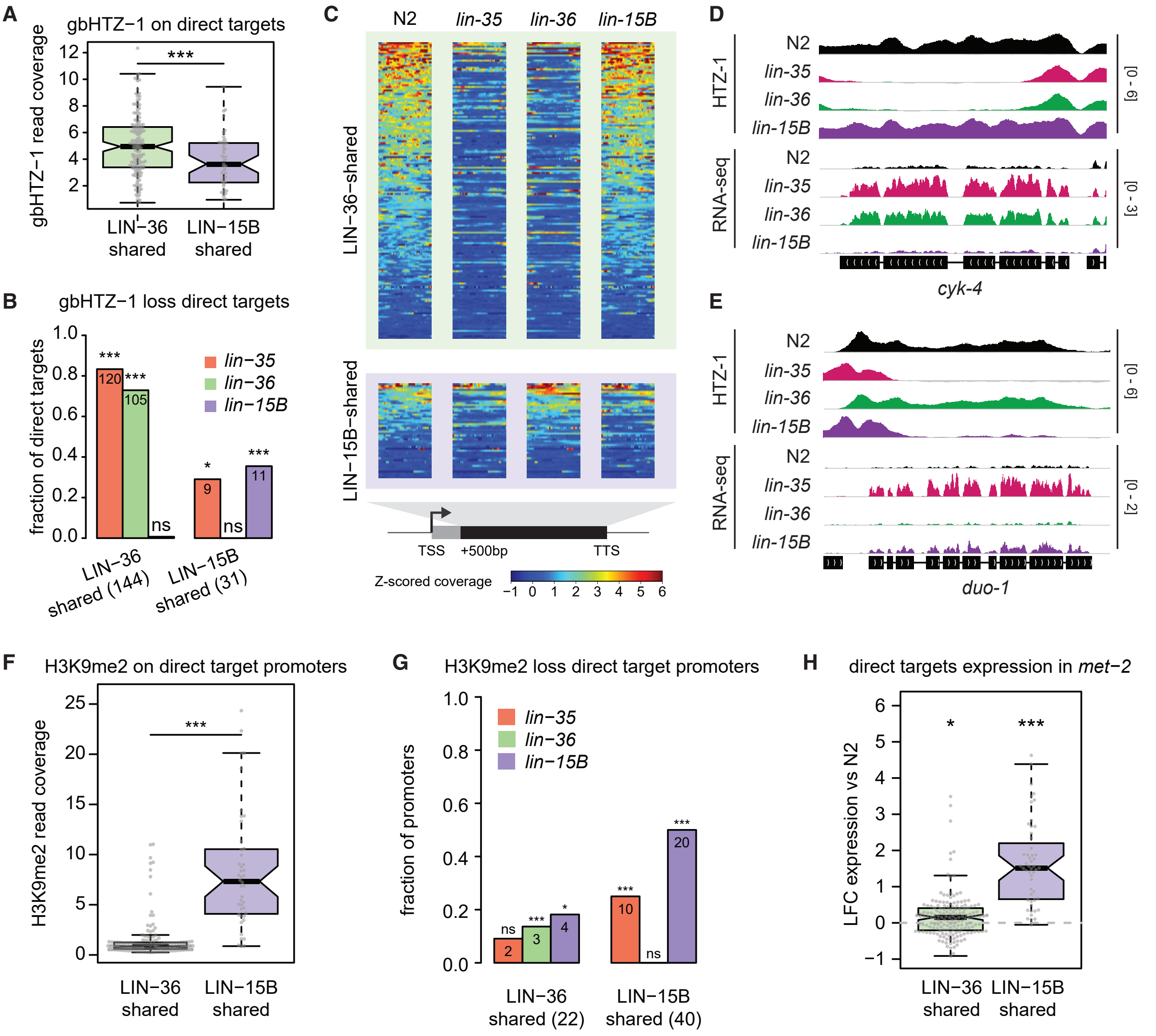

Figure 2. Gene body HTZ-1 and promoter H3K9me2 at LIN-36-shared and LIN-15B-shared targets

(A) HTZ-1 ChIP-seq coverage over gene bodies of LIN-36-shared and LIN-15B-shared targets. ***p < 0.001, Wilcoxon rank sum test.

(B) Fraction (and number) of LIN-36-shared and LIN-15B-shared direct targets showing a significant loss of gbHTZ-1 in the respective mutants. *p < 0.05, **p < 0.01, and ***p < 0.001, overrepresentation of gbHTZ-1 loss by hypergeometric test with Benjamini-Hochberg (BH) correction.

(C) gbHTZ-1 ChIP-seq coverage in wild type, *lin-35*, *lin-36*, and *lin-15B* mutants over LIN-36-shared and LIN-15B-shared direct targets.

(D and E) IGV view of HTZ-1 ChIP-seq (BEADS-normalized coverage) and RNA-seq (RPM) profiles over a LIN-36-shared (D) and LIN-15B-shared (E) direct target.

(F) H3K9me2 ChIP-seq coverage at promoters of LIN-36-shared and LIN-15B-shared targets, respectively. ***p < 0.001, Wilcoxon rank sum test.

(G) Fraction (and number) of LIN-36-shared and LIN-15B-shared direct target promoters showing a significant loss of H3K9me2 in the respective mutants. *p < 0.05 and ***p < 0.001, overrepresentation of gbHTZ-1 loss by hypergeometric test with BH correction.

(H) Log2-fold change of LIN-36-shared and LIN-15B-shared target expression between *met-2* mutant and wild type. ***p < 0.001 and *p < 0.05, t test.

similarities between the *lin-36* and *efl-1* transcriptomes suggest that EFL-1 functions as a transcriptional repressor specifically at LIN-36-shared DREAM targets.

Motifs at LIN-36-shared and LIN-15B-shared targets

To investigate the nature of the differential regulation of the LIN-36-shared versus LIN-15B-shared targets, we searched for DNA sequence motifs that might distinguish their respective promoters (see STAR Methods). E2F motifs and sequences similar to cell-cycle-dependent element (CDE) cell-cycle homology region (CHR) motifs were previously observed to be enriched at DREAM target regions (Kirienko and Fay, 2007; Latorre et al., 2015; Tabuchi et al., 2011). CDE-CHR is a bipartite motif in which the CDE has an E2F consensus (Müller and Engeland, 2010). In line with

CellPress
OPEN ACCESS

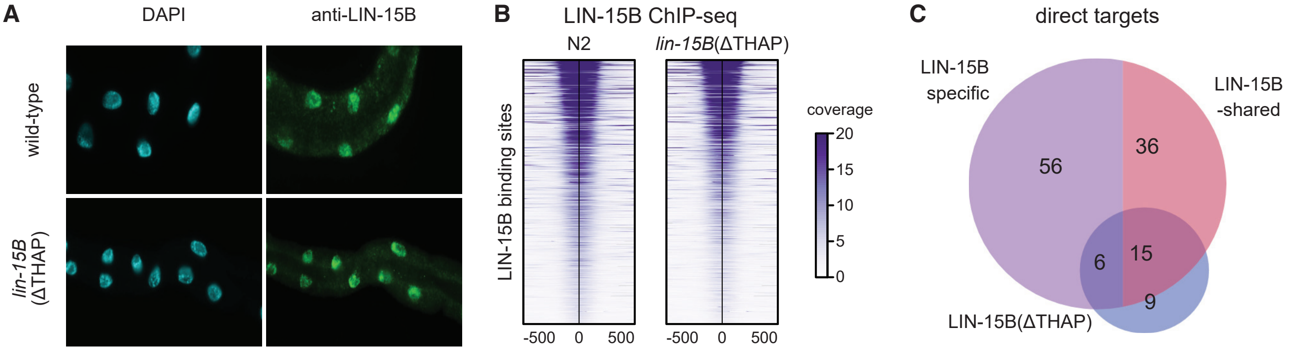

Figure 3. LIN-36 and LIN-15B require their THAP domains for proper function

(A) Immunofluorescence of LIN-15B in wild type and *lin-15B(ΔTHAP)* mutant.

(B) Heatmap of BEADS-normalized LIN-15B ChIP-seq coverage in wild type and *lin-15B(ΔTHAP)* mutant centered over wild-type LIN-15B peaks.

(C) Venn diagram of the overlap between direct targets in the indicated mutants. Direct targets shared with LIN-36 were excluded from the total count.

previous studies, we confirmed CDE-CHR and E2F among the most enriched motifs when searching the sets of all LIN-15B, LIN-36, LIN-35, or EFL-1 peaks (Table S2; Figure S4A). As expected from the high degree of overlap among the four peak sets, the E2F, CDE-CHR, and several other motifs found are very similar to each other; none showed differential enrichment in LIN-36-shared versus LIN-15B-shared target sites (Table S2).

To further investigate potential sequence differences in the LIN-36-shared versus LIN-15B-shared targets, we searched for DNA sequence motifs in the two sets of direct target sequences (see STAR Methods). We found variants of the E2F motif in LIN-36-shared targets (E2F-a1 and E2F-a2) and LIN-15B-shared targets (E2F-b), as well as a previously undescribed motif (here called LONG-a and LONG-b) (Table S2; Figure S4B). E2F-a1 closely matches the E2F motifs found in the searches of all LIN-15B, LIN-36, LIN-35, or EFL-1 peaks (Table S2). Notably, we observed that both the E2F-b and the LONG motifs show stronger association with LIN-15B-shared targets compared to LIN-36-shared targets (Figure S4C). E2F-a1 and E2F-a2 showed a nonsignificant trend for enrichment in LIN-36-shared targets. Almost all direct target peaks contained one or more sites corresponding to an E2F, CDE-CHR, or LONG motif, but we did not observe any significantly distinct motif combinations in LIN-15B-shared or LIN-36-shared targets (Figures S4D and S4E). We note that the small number of direct target peaks may have limited our ability to identify preferentially associated motifs. Nonetheless, the observed differences in E2F and LONG motifs might explain in part the distinct regulation of LIN-15B-shared and LIN-36-shared target genes.

Requirements for the LIN-36 and LIN-15B THAP domains

LIN-36 and LIN-15B both harbor a THAP domain (Figure S5A). To assess the requirements for the THAP domains, we created in-frame deletion alleles that removed THAP domain sequence from the endogenous *lin-36* and *lin-15B* genes (Figure S5A). We found that LIN-36(ΔTHAP) was not detectable by western blot or immunofluorescence, suggesting it is needed for LIN-36 stability (Figures S5B and S5C). In line with this, gene expression changes in *lin-36(ΔTHAP)* mutants are similar to those of the full-deletion mutant (Figure S5D; Table S3). As LIN-36(ΔTHAP) was not detectable, its activity could not be assessed.

We found that the LIN-15B(ΔTHAP) mutant protein localized to the nucleus similar to the endogenous protein (Figure 3A). LIN-15B(ΔTHAP) also displayed a ChIP-binding pattern similar to that of the wild-type protein, with 6774/8861 (~76%) LIN-15B peaks found in wild type also observed in *lin-15B(ΔTHAP)* (Figures 3B; Table S2). Despite the relatively normal localization pattern, 160 genes were derepressed in *lin-15B(ΔTHAP)* mutants, including 29% of LIN-15B-shared targets, all of which retained LIN-15B(ΔTHAP) binding (Figures 3C and S5E; Table S3). We conclude that the LIN-15B THAP domain is not essential for binding to its targets, but it is important for LIN-15B function. The finding that LIN-15B(ΔTHAP) localizes to LIN-15B sites suggests its recruitment is mediated either by other putative DNA-binding domains (Figure S5A; Table S1) or by binding to other factors.

LIN-36 and LIN-35 co-facilitate binding, whereas LIN-15B and LIN-35 mutually inhibit binding

To investigate potential interdependencies in chromatin binding at the LIN-36- and LIN-15B-specific targets, we conducted ChIP-seq analyses in mutants (Table S4). We found that LIN-35 and LIN-36 promote the association of EFL-1 and each other at LIN-36-shared targets, with >50% of sites dropping in signal in *lin-36* and *lin-35* mutants (Figures 4A and 4B, left panels). In contrast, LIN-36-shared targets showed normal levels of LIN-35, LIN-36, and EFL-1 in *lin-15B* mutants, consistent with the lack of requirement for LIN-15B at these targets (Figure 4C, left panel). We also found that LIN-15B binding at LIN-36-shared targets was independent of LIN-36 (Figure 4A, left panel). Therefore, LIN-35 and LIN-36 appear to mutually facilitate complex formation and/or stability at LIN-36-shared targets.

The LIN-15B-shared targets are strikingly different. At many of these sites, the loss of LIN-15B resulted in an unexpected increase of LIN-35, LIN-36, and EFL-1 signals (Figure 4C, right panels). Similarly, *lin-35* mutants showed a significant increase in LIN-15B occupancy at LIN-15B-shared targets (Figure 4B, right panels). In contrast, loss of LIN-36 caused only minor, mostly not significant differences in LIN-35, LIN-15B, and EFL-1 binding, (Figure 4A, right panel). Intriguingly, we found

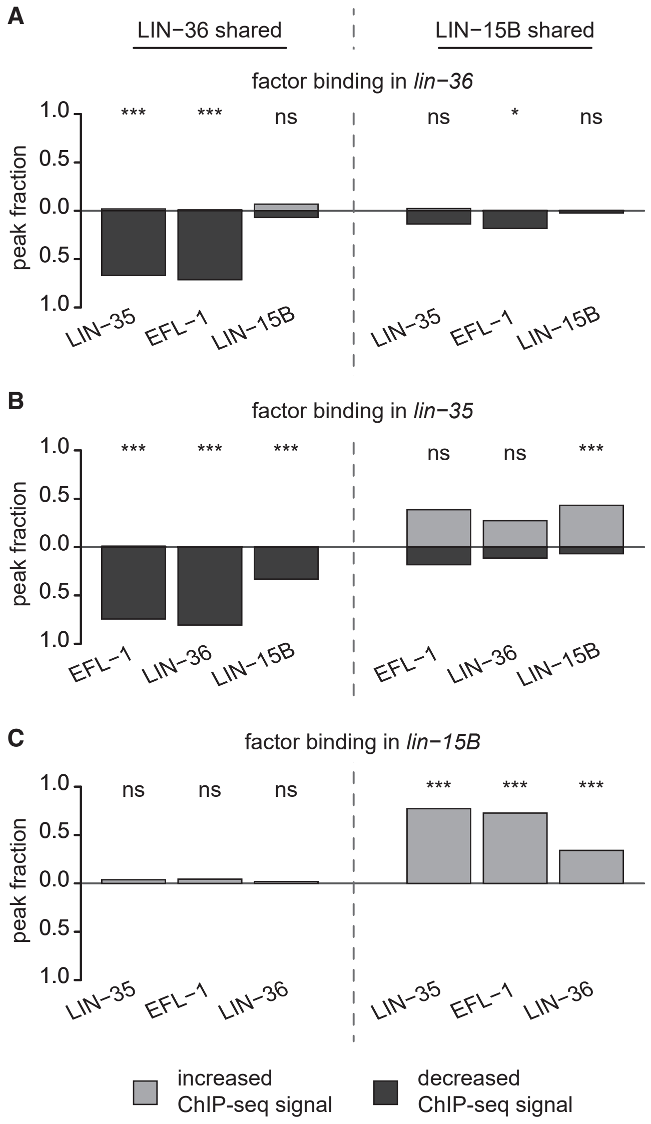

Figure 4. LIN-36 and LIN-35 facilitate, whereas LIN-15B and LIN-35 mutually inhibit, each other's binding

(A–C) Fraction of LIN-36-shared (left) and LIN-15B-shared (right) promoter-associated peaks showing a significant increase or decrease in ChIP-seq signal in *lin-36* (A), *lin-35* (B), and *lin-15B* (C) mutants compared to wild type. ***$p < 0.001$; *$p < 0.05$; ns, $p > 0.05$; Fisher's exact test with BH correction.

that the strength of LIN-15B(ΔTHAP) binding was significantly increased at ~38% of LIN-15B-shared targets (Figures S5F and S5G), suggesting that the THAP domain may destabilize LIN-15B binding. The finding that LIN-35 and LIN-15B repress LIN-15B-shared targets while mutually antagonizing chromatin association suggests a potential dynamic cycling of DREAM and LIN-15B, which may involve the LIN-15B THAP domain.

DISCUSSION

The DREAM complex represses cell-cycle genes to enforce cellular quiescence and represses developmental genes to ensure correct patterns of gene expression. While the roles of DREAM have been described in different animals, its mechanism of action is still unclear. Here, we show that two THAP domain proteins, LIN-36 and LIN-15B, act with DREAM to repress different sets of target genes through distinct mechanisms.

We found that LIN-36 and LIN-15B bind to thousands of genomic sites shared with LIN-35/Rb. Despite the similarity in binding patterns, genes derepressed upon loss of LIN-36 and LIN-15B are mostly distinct. Consistent with our finding that direct LIN-36 targets are highly enriched for cell-cycle functions, previous work has highlighted a role for LIN-36 in the *lin-35* pathway to prevent S-phase entry (Boxem and van den Heuvel, 2002). We also found that the E2F protein and DREAM component EFL-1 is required for repression of LIN-36 shared targets, but not for LIN-15B-shared targets. Through mutant analyses, we found that LIN-36 and DREAM mutually stabilize their chromatin association at shared direct targets, and both facilitate high levels of H2A variant HTZ-1 on gene bodies, which we previously found exerts a repressive role on gene expression (Latorre et al., 2015).

The targets that LIN-15B represses with DREAM largely have germline-specific expression. In starved L1 larvae, which are essentially comprised of somatic cells, the promoters of LIN-15B-shared targets have a closed chromatin environment and high levels of H3K9me2. LIN-15B, LIN-35/Rb, and the histone methyltransferase MET-2 are required for H3K9me2 marking, and MET-2 is required for the repression of many LIN-15B-shared targets. LIN-35 and LIN-15B ChIP signal is considerably lower at these targets than at LIN-36-shared targets. In contrast to the mutual dependence of LIN-36 and DREAM, LIN-15B and DREAM appear to destabilize each other at shared target promoters. We suggest that the dynamic cycling resulting from the mutual destabilization of LIN-15B and DREAM factors may facilitate MET-2 access at LIN-15B-shared targets promoters, thus enabling their repression through deposition of repressive H3K9me2.

The presence of a THAP domain in both LIN-36 and LIN-15B suggests a special relationship between DREAM- and THAP-domain-containing proteins. In support of this idea, the human Rb protein shares targets with the THAP1 protein, whose ectopic expression inhibits proliferation in primary human endothelial cells through the transcriptional repression of E2F/Rb targets (Cayrol et al., 2007). Moreover, endogenous THAP1 is necessary for proliferation, suggesting that optimal THAP1 levels are critical. The human THAP11 protein has also been implicated in the regulation of E2F targets and cell proliferation, although its activity is mediated by the interaction with other factors (Parker et al., 2014). The lack of clear conservation of THAP domain proteins outside this domain suggests that the THAP domain may mediate interactions with DREAM complex. Future work in different systems will further clarify the mechanisms of gene repression employed by the THAP domain protein/DREAM network.

STAR★METHODS

Detailed methods are provided in the online version of this paper and include the following:

- KEY RESOURCES TABLE
- RESOURCE AVAILABILITY

- Lead contact
- Materials availability
- Data and code availability
- EXPERIMENTAL MODEL AND SUBJECT DETAILS
  - Worm culture and strains
  - Generation of psep-1::his-58::eGFP, lin-36::eGFP, lin-36 deletion, and THAP domain deletion alleles
- METHOD DETAILS
  - RNAi Screen
  - RNAi screen of THAP genes
  - Collection of starved L1 animals for RNA-seq and ChIP-seq
  - ChIP-seq
  - RNA-seq
  - DNA binding domain prediction
  - Data processing
  - Differential expression analysis
  - Peak calls and annotation to genes
  - Identification of direct targets
  - GO enrichment analysis
  - Gene body HTZ-1 enrichment
  - H3K9me2 enrichment
  - Motif enrichment analysis
  - Identification of differentially bound peaks
  - Graphical abstract
- QUANTIFICATION AND STATISTICAL ANALYSIS

SUPPLEMENTAL INFORMATION

Supplemental information can be found online at https://doi.org/10.1016/j.celrep.2021.109835.

ACKNOWLEDGMENTS

The work was supported by an SNF Fellowship to F.N.C. (P400PB_180795) and a Wellcome Senior Research Fellowship (101863) and MRC Programme grant (MR/S021620/1) to J.A. We also acknowledge core support from Wellcome (092096) and Cancer Research UK (C6946/A14492).

AUTHOR CONTRIBUTIONS

Conceptualization and methodology, C.G. and J.A.; software and formal analysis, C.G., F.N.C., and N.H.; investigation, C.G., F.N.C., A.A., C.C., Y.D., A.F., and J.M.; writing – original draft preparation, C.G., F.N.C., and J.A.; supervision and funding acquisition, J.A.

DECLARATION OF INTERESTS

The authors declare no competing interests.

Received: August 28, 2020
Revised: June 3, 2021
Accepted: September 24, 2021
Published: October 19, 2021

REFERENCES

Angeles-Albores, D., Lee, R., Chan, J., and Sternberg, P. (2018). Two new functions in the WormBase Enrichment Suite. MicroPubl. Biol., 25–2.

Argmann, C.A., Dierich, A., and Auwerx, J. (2006). Uses of forward and reverse genetics in mice to study gene function. Curr. Protoc. Mol. Biol. *Chapter 29*, 1–43.

Arribere, J.A., Bell, R.T., Fu, B.X.H., Artiles, K.L., Hartman, P.S., and Fire, A.Z. (2014). Efficient marker-free recovery of custom genetic modifications with CRISPR/Cas9 in Caenorhabditis elegans. Genetics *198*, 837–846.

Bailey, T.L., Boden, M., Buske, F.A., Frith, M., Grant, C.E., Clementi, L., Ren, J., Li, W.W., and Noble, W.S. (2009). MEME SUITE: tools for motif discovery and searching. Nucleic Acids Res. *37*, W202-8.

Bainor, A.J., Saini, S., Calderon, A., Casado-Polanco, R., Giner-Ramirez, B., Moncada, C., Cantor, D.J., Ernlund, A., Litovchick, L., and David, G. (2018). The HDAC-Associated Sin3B Protein Represses DREAM Complex Targets and Cooperates with APC/C to Promote Quiescence. Cell Rep. *25*, 2797–2807.e8.

Balakrishnan, M.P., Cilenti, L., Mashak, Z., Popat, P., Alnemri, E.S., and Zervos, A.S. (2009). THAP5 is a human cardiac-specific inhibitor of cell cycle that is cleaved by the proapoptotic Omi/HtrA2 protease during cell death. Am. J. Physiol. Heart Circ. Physiol. *297*, H643–H653.

Bessler, J.B., Andersen, E.C., and Villeneuve, A.M. (2010). Differential localization and independent acquisition of the H3K9me2 and H3K9me3 chromatin modifications in the Caenorhabditis elegans adult germ line. PLoS Genet. *6*, e1000830.

Boxem, M., and van den Heuvel, S. (2002). C. elegans class B synthetic multivulva genes act in G(1) regulation. Curr. Biol. *12*, 906–911.

Brenner, S. (2003). Nature's gift to science (Nobel lecture). ChemBioChem *4*, 683–687.

Cao, J., Packer, J.S., Ramani, V., Cusanovich, D.A., Huynh, C., Daza, R., Qiu, X., Lee, C., Furlan, S.N., Steemers, F.J., et al. (2017). Comprehensive single-cell transcriptional profiling of a multicellular organism. Science *357*, 661–667.

Cayrol, C., Lacroix, C., Mathe, C., Ecochard, V., Ceribelli, M., Loreau, E., Lazar, V., Dessen, P., Mantovani, R., Aguilar, L., and Girard, J.P. (2007). The THAP-zinc finger protein THAP1 regulates endothelial cell proliferation through modulation of pRB/E2F cell-cycle target genes. Blood *109*, 584–594.

Ceron, J., Rual, J.-F., Chandra, A., Dupuy, D., Vidal, M., and van den Heuvel, S. (2007). Large-scale RNAi screens identify novel genes that interact with the C. elegans retinoblastoma pathway as well as splicing-related components with synMuv B activity. BMC Dev. Biol. *7*, 30.

Chesney, M.A., Kidd, A.R., 3rd, and Kimble, J. (2006). gon-14 functions with class B and class C synthetic multivulva genes to control larval growth in Caenorhabditis elegans. Genetics *172*, 915–928.

Cheung, M.-S., Down, T.A., Latorre, I., and Ahringer, J. (2011). Systematic bias in high-throughput sequencing data and its correction by BEADS. Nucleic Acids Res. *39*, e103.

Clouaire, T., Roussigne, M., Ecochard, V., Mathe, C., Amalric, F., and Girard, J.-P. (2005). The THAP domain of THAP1 is a large C2CH module with zinc-dependent sequence-specific DNA-binding activity. Proc. Natl. Acad. Sci. USA *102*, 6907–6912.

Dick, F.A., and Rubin, S.M. (2013). Molecular mechanisms underlying RB protein function. Nat. Rev. Mol. Cell Biol. *14*, 297–306.

Dobin, A., Davis, C.A., Schlesinger, F., Drenkow, J., Zaleski, C., Jha, S., Batut, P., Chaisson, M., and Gingeras, T.R. (2013). STAR: ultrafast universal RNA-seq aligner. Bioinformatics *29*, 15–21.

Du, W., Vidal, M., Xie, J.E., and Dyson, N. (1996). RBF, a novel RB-related gene that regulates E2F activity and interacts with cyclin E in Drosophila. Genes Dev. *10*, 1206–1218.

Eddy, S.R. (1998). Profile hidden Markov models. Bioinformatics *14*, 755–763.

Fraser, A.G., Kamath, R.S., Zipperlen, P., Martinez-Campos, M., Sohrmann, M., and Ahringer, J. (2000). Functional genomic analysis of C. elegans chromosome I by systematic RNA interference. Nature *408*, 325–330.

Frøkjaer-Jensen, C., Davis, M.W., Hopkins, C.E., Newman, B.J., Thummel, J.M., Olesen, S.-P., Grunnet, M., and Jorgensen, E.M. (2008). Single-copy insertion of transgenes in Caenorhabditis elegans. Nat. Genet. *40*, 1375–1383.

Gervais, V., Campagne, S., Durand, J., Muller, I., and Milon, A. (2013). NMR studies of a new family of DNA binding proteins: the THAP proteins. J. Biomol. NMR *56*, 3–15.

Goetsch, P.D., Garrigues, J.M., and Strome, S. (2017). Loss of the Caenorhabditis elegans pocket protein LIN-35 reveals MuvB's innate function as the repressor of DREAM target genes. PLoS Genet. *13*, e1007088.

Haeussler, M., Schönig, K., Eckert, H., Eschstruth, A., Mianné, J., Renaud, J.-B., Schneider-Maunoury, S., Shkumatava, A., Teboul, L., Kent, J., et al. (2016). Evaluation of off-target and on-target scoring algorithms and integration into the guide RNA selection tool CRISPOR. Genome Biol. *17*, 148.

Hauser, S., Ulrich, T., Wurster, S., Schmitt, K., Reichert, N., and Gaubatz, S. (2012). Loss of LIN9, a member of the DREAM complex, cooperates with SV40 large T antigen to induce genomic instability and anchorage-independent growth. Oncogene *31*, 1859–1868.

Hubley, R., Finn, R.D., Clements, J., Eddy, S.R., Jones, T.A., Bao, W., Smit, A.F.A., and Wheeler, T.J. (2016). The Dfam database of repetitive DNA families. Nucleic Acids Res. *44* (*D1*), D81–D89.

Jänes, J., Dong, Y., Schoof, M., Serizay, J., Appert, A., Cerrato, C., Woodbury, C., Chen, R., Gemma, C., Huang, N., et al. (2018). Chromatin accessibility dynamics across *C. elegans* development and ageing. eLife *7*, 1–24.

Kamath, R.S., Fraser, A.G., Dong, Y., Poulin, G., Durbin, R., Gotta, M., Kanapin, A., Le Bot, N., Moreno, S., Sohrmann, M., et al. (2003). Systematic functional analysis of the Caenorhabditis elegans genome using RNAi. Nature *421*, 231–237.

Kirienko, N.V., and Fay, D.S. (2007). Transcriptome profiling of the C. elegans Rb ortholog reveals diverse developmental roles. Dev. Biol. *305*, 674–684.

Korenjak, M., Taylor-Harding, B., Binné, U.K., Satterlee, J.S., Stevaux, O., Aasland, R., White-Cooper, H., Dyson, N., and Brehm, A. (2004). Native E2F/RBF complexes contain Myb-interacting proteins and repress transcription of developmentally controlled E2F target genes. Cell *119*, 181–193.

Latorre, I., Chesney, M.A., Garrigues, J.M., Stempor, P., Appert, A., Francesconi, M., Strome, S., and Ahringer, J. (2015). The DREAM complex promotes gene body H2A.Z for target repression. Genes Dev. *29*, 495–500.

Lee, E.Y.-H.P., Chang, C.-Y., Hu, N., Wang, Y.-C.J., Lai, C.-C., Herrup, K., Lee, W.-H., and Bradley, A. (1992). Mice deficient for Rb are nonviable and show defects in neurogenesis and haematopoiesis. Nature *359*, 288–294.

Lewis, P.W., Beall, E.L., Fleischer, T.C., Georlette, D., Link, A.J., and Botchan, M.R. (2004). Identification of a Drosophila Myb-E2F2/RBF transcriptional repressor complex. Genes Dev. *18*, 2929–2940.

Lex, A., Gehlenborg, N., Strobelt, H., Vuillemot, R., and Pfister, H. (2014). UpSet: Visualization of intersecting sets. IEEE Trans. Vis. Comput. Graph. *20*, 1983–1992.

Li, H., and Durbin, R. (2010). Fast and accurate long-read alignment with Burrows-Wheeler transform. Bioinformatics *26*, 589–595.

Li, H., Handsaker, B., Wysoker, A., Fennell, T., Ruan, J., Homer, N., Marth, G., Abecasis, G., and Durbin, R.; 1000 Genome Project Data Processing Subgroup (2009). The Sequence Alignment/Map format and SAMtools. Bioinformatics *25*, 2078–2079.

Li, Q., Brown, J.B., Huang, H., and Bickel, P.J. (2011). Measuring reproducibility of high-throughput experiments. Ann. Appl. Stat. *5*, 1752–1779.

Litovchick, L., Sadasivam, S., Florens, L., Zhu, X., Swanson, S.K., Velmurugan, S., Chen, R., Washburn, M.P., Liu, X.S., and DeCaprio, J.A. (2007). Evolutionarily conserved multisubunit RBL2/p130 and E2F4 protein complex represses human cell cycle-dependent genes in quiescence. Mol. Cell *26*, 539–551.

Litovchick, L., Florens, L.A., Swanson, S.K., Washburn, M.P., and DeCaprio, J.A. (2011). DYRK1A protein kinase promotes quiescence and senescence through DREAM complex assembly. Genes Dev. *25*, 801–813.

Liu, Z., Gersbach, E., Zhang, X., Xu, X., Dong, R., Lee, P., Liu, J., Kong, B., Shao, C., and Wei, J.J. (2013). miR-106a represses the Rb tumor suppressor p130 to regulate cellular proliferation and differentiation in high-grade serous ovarian carcinoma. Mol. Cancer Res. *11*, 1314–1325.

Love, M.I., Huber, W., and Anders, S. (2014). Moderated estimation of fold change and dispersion for RNA-seq data with DESeq2. Genome Biol. *15*, 550.

Lu, X., and Horvitz, H.R. (1998). lin-35 and lin-53, two genes that antagonize a C. elegans Ras pathway, encode proteins similar to Rb and its binding protein RbAp48. Cell *95*, 981–991.

Malumbres, M., and Barbacid, M. (2009). Cell cycle, CDKs and cancer: a changing paradigm. Nat. Rev. Cancer *9*, 153–166.

Müller, G.A., and Engeland, K. (2010). The central role of CDE/CHR promoter elements in the regulation of cell cycle-dependent gene transcription. FEBS J. *277*, 877–893.

Narasimhan, K., Lambert, S.A., Yang, A.W.H., Riddell, J., Mnaimneh, S., Zheng, H., Albu, M., Najafabadi, H.S., Reece-Hoyes, J.S., Fuxman Bass, J.I., et al. (2015). Mapping and analysis of Caenorhabditis elegans transcription factor sequence specificities. eLife *4*, 1–53.

Nor Rashid, N., Yusof, R., and Watson, R.J. (2011). Disruption of repressive p130-DREAM complexes by human papillomavirus 16 E6/E7 oncoproteins is required for cell-cycle progression in cervical cancer cells. J. Gen. Virol. *92*, 2620–2627.

Ouellet, J., and Roy, R. (2007). The lin-35/Rb and RNAi pathways cooperate to regulate a key cell cycle transition in C. elegans. BMC Dev. Biol. *7*, 38.

Page, B.D., Guedes, S., Waring, D., and Priess, J.R. (2001). The C. elegans E2F- and DP-related proteins are required for embryonic asymmetry and negatively regulate Ras/MAPK signaling. Mol. Cell *7*, 451–460.

Paix, A., Folkmann, A., Rasoloson, D., and Seydoux, G. (2015). High Efficiency, Homology-Directed Genome Editing in Caenorhabditis elegans Using CRISPR-Cas9 Ribonucleoprotein Complexes. Genetics *201*, 47–54.

Paix, A., Folkmann, A., Goldman, D.H., Kulaga, H., Grzelak, M.J., Rasoloson, D., Paidemarry, S., Green, R., Reed, R.R., and Seydoux, G. (2017). Precision genome editing using synthesis-dependent repair of Cas9-induced DNA breaks. Proc. Natl. Acad. Sci. USA *114*, E10745.

Parker, J.B., Yin, H., Vinckevicius, A., and Chakravarti, D. (2014). Host cell factor-1 recruitment to E2F-bound and cell-cycle-control genes is mediated by THAP11 and ZNF143. Cell Rep. *9*, 967–982.

Poulin, G., Dong, Y., Fraser, A.G., Hopper, N.A., and Ahringer, J. (2005). Chromatin regulation and sumoylation in the inhibition of Ras-induced vulval development in Caenorhabditis elegans. EMBO J. *24*, 2613–2623.

Quinlan, A.R., and Hall, I.M. (2010). BEDTools: a flexible suite of utilities for comparing genomic features. Bioinformatics *26*, 841–842.

R Development Core Team (2011). R: A Language and Environment for Statistical Computing (Springer Berlin Heidelberg).

Ramírez, F., Ryan, D.P., Grüning, B., Bhardwaj, V., Kilpert, F., Richter, A.S., Heyne, S., Dündar, F., and Manke, T. (2016). deepTools2: a next generation web server for deep-sequencing data analysis. Nucleic Acids Res. *44* (*W1*), W160-5.

Rayman, J.B., Takahashi, Y., Indjeian, V.B., Dannenberg, J.H., Catchpole, S., Watson, R.J., te Riele, H., and Dynlacht, B.D. (2002). E2F mediates cell cycle-dependent transcriptional repression in vivo by recruitment of an HDAC1/mSin3B corepressor complex. Genes Dev. *16*, 933–947.

Rechtsteiner, A., Costello, M.E., Egelhofer, T.A., Garrigues, J.M., Strome, S., and Petrella, L.N. (2019). Repression of Germline Genes in *Caenorhabditis elegans* Somatic Tissues by H3K9 Dimethylation of Their Promoters. Genetics *212*, 125–140.

Reddy, K.C., and Villeneuve, A.M. (2004). C. elegans HIM-17 links chromatin modification and competence for initiation of meiotic recombination. Cell *118*, 439–452.

Reichert, N., Wurster, S., Ulrich, T., Schmitt, K., Hauser, S., Probst, L., Götz, R., Ceteci, F., Moll, R., Rapp, U., and Gaubatz, S. (2010). Lin9, a subunit of the mammalian DREAM complex, is essential for embryonic development, for survival of adult mice, and for tumor suppression. Mol. Cell. Biol. *30*, 2896–2908.

Richter, A., Hollstein, R., Hebert, E., Vulinovic, F., Eckhold, J., Osmanovic, A., Depping, R., Kaiser, F.J., and Lohmann, K. (2017). In-depth Characterization of the Homodimerization Domain of the Transcription Factor THAP1 and Dystonia-Causing Mutations Therein. J. Mol. Neurosci. *62*, 11–16.

Roussigne, M., Kossida, S., Lavigne, A.-C., Clouaire, T., Ecochard, V., Glories, A., Amalric, F., and Girard, J.-P. (2003). The THAP domain: a novel protein motif with similarity to the DNA-binding domain of P element transposase. Trends Biochem. Sci. *28*, 66–69.

CellPress
OPEN ACCESS

Rual, J.-F., Ceron, J., Koreth, J., Hao, T., Nicot, A.-S., Hirozane-Kishikawa, T., Vandenhaute, J., Orkin, S.H., Hill, D.E., van den Heuvel, S., and Vidal, M. (2004). Toward improving Caenorhabditis elegans phenome mapping with an ORFeome-based RNAi library. Genome Res. *14* (*10B*), 2162–2168.

Sadasivam, S., and DeCaprio, J.A. (2013). The DREAM complex: master coordinator of cell cycle-dependent gene expression. Nat. Rev. Cancer *13*, 585–595.

Saito, R.M., Perreault, A., Peach, B., Satterlee, J.S., and van den Heuvel, S. (2004). The CDC-14 phosphatase controls developmental cell-cycle arrest in C. elegans. Nat. Cell Biol. *6*, 777–783.

Schade, A.E., Oser, M.G., Nicholson, H.E., and DeCaprio, J.A. (2019). Cyclin D-CDK4 relieves cooperative repression of proliferation and cell cycle gene expression by DREAM and RB. Oncogene *38*, 4962–4976.

Schmit, F., Korenjak, M., Mannefeld, M., Schmitt, K., Franke, C., von Eyss, B., Gagrica, S., Hänel, F., Brehm, A., and Gaubatz, S. (2007). LINC, a human complex that is related to pRB-containing complexes in invertebrates regulates the expression of G2/M genes. Cell Cycle *6*, 1903–1913.

Stiernagle, T. (2006). Maintenance of C. elegans. WormBook, 1–11.

Tabuchi, T.M., Deplancke, B., Osato, N., Zhu, L.J., Barrasa, M.I., Harrison, M.M., Horvitz, H.R., Walhout, A.J.M., and Hagstrom, K.A. (2011). Chromosome-biased binding and gene regulation by the Caenorhabditis elegans DRM complex. PLoS Genet. *7*, e1002074.

Zeiser, E., Frøkjær-Jensen, C., Jorgensen, E., and Ahringer, J. (2011). MosSCI and gateway compatible plasmid toolkit for constitutive and inducible expression of transgenes in the C. elegans germline. PLoS ONE *6*, e20082.

Zhang, Y., Liu, T., Meyer, C.A., Eeckhoute, J., Johnson, D.S., Bernstein, B.E., Nusbaum, C., Myers, R.M., Brown, M., Li, W., and Liu, X.S. (2008). Model-based analysis of ChIP-Seq (MACS). Genome Biol. *9*, R137.

STAR★METHODS

KEY RESOURCES TABLE

REAGENT or RESOURCE	SOURCE	IDENTIFIER
Antibodies		
anti-GFP	Abcam	Cat# ab290, RRID:**AB_303395**
anti-H3K9me2	Wako	Cat# 302-32369
anti-HTZ-1	Latorre et al., 2015	JA00001 (SK2088_SK2089)
anti-LIN-35	This study	Q2001
anti-EFL-1	Latorre et al., 2015	Q3590
anti-LIN-15B	Rechtsteiner et al., 2019	Q2330
Bacterial and virus strains		
Escherichia coli: OP50	Caenorhabditis Genetics Center	N/A
Escherichia coli HT115(DE3)	Caenorhabditis Genetics Center	N/A
C. elegans RNAi feeding libraries	Fraser et al., 2000; Kamath et al., 2003; Rual et al., 2004	N/A
Chemicals, peptides, and recombinant proteins		
TriPure	Roche	Cat# 11667157001
Critical commercial assays		
Protein G Dynabeads	Invitrogen	Cat# 10446293
TruSeq RNA Library Prep Kit v2	Illumina	Cat# RS-122-2002
AMPure XP beads	Beckman	Cat# A63881
Protein A Dynabeads	Invitrogen	Cat# 10334693
Deposited data		
Raw and analyzed data	This paper	GEO: GSE155191
Experimental models: Organisms/strains		
C. elegans: Strain: JA1850 *lin-36(we36)*	This study	N/A
C. elegans: N2 (Bristol), Wild type	Caenorhabditis Genetics Center	N/A
C. elegans: Strain: JA1798 lin-15B(we23)	This study	N/A
C. elegans: Strain: JA1810 *lin-36(we30[lin-36::eGFP])*	This study	N/A
C. elegans: Strain: JA1811 *lin-36(we31)*	This study	N/A
C. elegans: Strain: MT13293 *met-2(n4256)*	Caenorhabditis Genetics Center	N/A
C. elegans: Strain: JA1697 unc-119(ed II); weSi118[psep-1::his-58::eGFP-tbb-2 3′UTR]	This study	N/A
C. elegans: Strain: JA1717 *unc-119(ed9); weSi118[psep-1::his-58::eGFP-tbb-2 3′UTR]; lin-35 (n745)*	This study	N/A
C. elegans: Strain: JA1821 *lin-36(we30[lin-36::eGFP]) III; lin-35(n745)*	This study	N/A
C. elegans: Strain: JA1819 *lin-36(we30[lin-36::eGFP]) III; lin-15B(n744)*	This study	N/A
C. elegans: Strain: MT8879 *dpl-1(n2994)*	Caenorhabditis Genetics Center	N/A
C. elegans: Strain: JJ1549 *efl-1(se1)*	Caenorhabditis Genetics Center	N/A
C. elegans: Strain: MT5470 *lin-37(n758)*	Caenorhabditis Genetics Center	N/A
C. elegans: Strain: JA1507 *lin-35(n745)*	Horvitz lab (outcrossed 5X)	N/A
C. elegans: Strain: MT2495 *lin-15B (n744)*	Caenorhabditis Genetics Center	N/A
C. elegans: Strain: MT6034 *lin-36(n766)*	Caenorhabditis Genetics Center	N/A

(*Continued on next page*)

CellPress
OPEN ACCESS

Continued

REAGENT or RESOURCE	SOURCE	IDENTIFIER
Software and algorithms		
yapc	Jänes et al., 2018	N/A
BEDTools v2.27.1	Quinlan and Hall, 2010	RRID:SCR_006646
deepTools 3.1.3	Ramírez et al., 2016	RRID:SCR_016366
STAR v2.5.4b	Dobin et al., 2013	RRID:SCR_004463
samtools v1.9	Li et al., 2009	RRID:SCR_002105
bwa v0.7.7	Li and Durbin, 2010	RRID:SCR_010910
macs2 v2.1.2	Zhang et al., 2008	RRID:SCR_013291
DESeq2 1.26.0	Love et al., 2014	RRID:SCR_015687
MEME 5.0.5	Bailey et al., 2009	RRID:SCR_001783
UpSetR	Lex et al., 2014	N/A
R 3.6	R Development Core Team, 2011	RRID:SCR_001905
HMMER 3.1b2	Eddy, 1998	RRID:SCR_005305
CRISPOR	Haeussler et al., 2016	RRID:SCR_015935
Biorender	biorender.com	RRID:SCR_018361

RESOURCE AVAILABILITY

Lead contact

Further information and requests for resources and reagents should be directed to and will be fulfilled by the Lead Contact, Julie Ahringer (ja219@cam.ac.uk).

Materials availability

All unique/stable reagents generated in this study are available from the lead contact without restriction.

Data and code availability

- RNA-seq and ChIP-seq datasets generated during this study are available at NCBI Gene Expression Omnibus (GEO) under accession code GSE155191.
- The code used for analyses is available in Data S1.
- Any additional information required to reanalyze the data reported in this work paper is available from the Lead Contact upon request.

EXPERIMENTAL MODEL AND SUBJECT DETAILS

Worm culture and strains

Strains were cultured using standard methods (Brenner, 2003). Strains used in the paper are given in the key resources table and Table S1.

Generation of psep-1::his-58::eGFP, lin-36::eGFP, lin-36 deletion, and THAP domain deletion alleles

The *psep-1::his-58::eGFP* transgene was generated using three-site Gateway cloning (Invitrogen) in the MosSCI compatible vector pCFJ150, which targets Mos site Mos1(ttTi5605) (Frøkjaer-Jensen et al., 2008). The *sep-1* promoter (chr I: 3439109-3438531) was cloned into site one. Plasmids pJA273 and pJA257 (Zeiser et al., 2011) were used to put *his-58* into site 2 and *eGFP::tbb-2-3′*UTR into site three, respectively. MosSCI lines were generated as described (Frøkjaer-Jensen et al., 2008).

CRISPR-Cas9 genome editing was used to generate the following strains: JA1798: *lin-15B(we23[ΔTHAP])* X, JA1810: *lin-36(we30[lin-36::eGFP])* III, JA1811: *lin-36(we30[lin-36::eGFP], we31[ΔTHAP])* III, and JA1850: *lin-36(we36) III* (Table S1). Injections were performed using gRNA-Cas9 ribonucleoprotein (RNP) complexes preassembled *in vitro* (Paix et al., 2017). *dpy-10* co-CRISPR method was used to enrich for desired edit (Arribere et al., 2014; Paix et al., 2015). Cas9 protein was made in-house (Paix et al., 2015); tracrRNA and crRNAs were purchased from Dharmacon or Integrated DNA Technologies; repair templates were purchased from IDT as Ultramer oligonucleotides; eGFP double stranded amplicons were generated by standard PCR (Paix et al., 2017). crRNAs were designed using the online CRISPOR tool (Haeussler et al., 2016). JA1798, JA1810 and JA1850 were made in the Bristol wild-type N2 background; JA1811 was made in JA1810.

METHOD DETAILS

RNAi Screen

An RNAi sub-library targeting 1104 known or predicted nuclear proteins was used for the RNAi (Table S1); RNAi clones were from (Fraser et al., 2000; Kamath et al., 2003) (Rual et al., 2004). The primary screen was carried out in four replicates, two feeding from the L3 stage and two feeding from the YA stage, the latter to avoid the high embryonic lethality induced by some clones. Bacteria were grown at 37°C overnight in 900 μL LB (supplemented with 10 μg/ml carbenicillin, 10 μg/ml tetracyline, and 100 U/ml nystatin) in 96 well plates. RNAi expression was induced through the addition of 4 mM IPTG, and bacteria were further incubated for 3 hours at 37°C. Bacteria were then pelleted and resuspended in 450 μL of S medium (Stiernagle, 2006), 50 μL was transferred into each well of a new 96-well plate, and approximately 10-15 L3 or YA *psep-1::his-58::eGFP* animals were placed into each well. The animals were monitored and when most had L1 progeny the L1s were analyzed for increased expression of the reporter using a COPAS (Union Biometrica) profiler by measuring fluorescence intensity of L1 sized progeny. In the primary screen, 210 clones induced de-repression of the reporter in two out of the four replicates and were included in four replicates of a secondary screen conducted using YA aninals. Of these, 36 showed de-repression in three out of four replicates and were considered to be hits (see Table S1). These clones were sequenced and verified.

RNAi screen of THAP genes

RNAi plates targeting THAP domain genes were prepared as in Argmann et al. (2006). Synchronized L3 *psep-1::his-58::eGFP* animals were placed onto RNAi plates and their progeny assessed daily for somatic GFP expression through visual observation under a fluorescent microscope, qualitatively compared to control RNAi. Experiments were carried out three times.

Collection of starved L1 animals for RNA-seq and ChIP-seq

Synchronized adults were grown at 20°C in liquid culture using standard S-basal medium and HB101 *E. coli*, bleached to isolate embryos, the eggs hatched 20-22 hours at 25°C in M9 buffer, and then the starved L1s were sucrose floated and collected by flash freezing in liquid nitrogen. The *efl-1(se1ts)* mutants were hatched at 26°C (Page et al., 2001).

ChIP-seq

Frozen starved L1 worms were ground to a powder, which was incubated in 1.5 mM EGS (Pierce 21565) in PBS for 8 minutes, followed by the addition of formaldehyde to a final concentration of 1%, and incubated for a further 8 minutes. The fixation was quenched for 5 minutes by the addition of 0.125 M glycine. Fixed tissue was washed 2X with PBS with protease inhibitors (Roche EDTA-free protease inhibitor cocktail tablets 05056489001) and once in FA buffer (50 mM HEPES pH7.5, 1 mM EDTA, 1% Triton X-100, 0.1% sodium deoxycholate, and 150 mM NaCl) with protease inhibitors (FA+), then resuspended in 1 mL FA+ buffer per 1 mL of ground worm powder. The extract was sonicated to an average size of ~250 base pairs using a Bioruptor Pico (Diagenode), and 10-20 ug of DNA was used per ChIP reaction, together with ~1ug DNA from *C. briggsae* ChIP extract. Antibodies used for ChIP are listed in the key resources table; the LIN-35 antibody Q2001 was raised by DNA immunisation (SDIX) against amino acids 72-171. ChIP-seq datasets are described in Table S5. ChIP and library preparations were done as described in Jänes et al. (2018).

RNA-seq

A single ball of frozen worms was used for RNA extractions. Total RNA was extracted using TriPure (Roche) and further purified using an RNeasy column (QIAGEN). RNA-seq libraries were prepared from 100-1000 ng of total RNA using the Illumina TruSeq RNA kit according to the manufacturers' instructions. RNA-seq datasets are given in Table S5.

DNA binding domain prediction

A list of putative DNA binding domains annotated in Pfam was obtained from Narasimhan et al. (2015). Each DBD model was mapped in LIN-15B and LIN-36 sequences using the hmmsearch tool from HMMER (Eddy, 1998).

Data processing

ChIP-seq and RNA-seq libraries were sequenced using Illumina HiSeq1500. ChIP-seq reads were aligned to a concatenated WS235/ce11 + cb3 assembly of the *C. elegans* and *C. briggsae* genomes using BWA v. 0.7.7 with default settings (BWA-backtrack algorithm) (Li and Durbin, 2010), but only *C. elegans* data were analyzed here. The SAMtools v. 0.1.19 'view' utility (Li et al., 2009) was used to convert the alignments to BAM format. Normalized mapq10 ChIP-seq coverage tracks were generated using the BEADS algorithm (Cheung et al., 2011). RNA-seq reads were aligned using STAR (Dobin et al., 2013) with the two-pass mode using the *C. elegans* gene annotation from Wormbase (version WS260) as a guide (after removing any gene annotation from the mitochondrial DNA). BigWig tracks were generated using the wigToBigWig tool downloaded from the UCSC website (https://hgdownload.soe.ucsc.edu/downloads.html). Processing of genomic coordinates was performed using the BEDTools suite (version 2.27.1) and in-house scripts. Statistical analyses were performed in R (R Development Core Team, 2011). Commands used to process data are available in Data S1.

Differential expression analysis

A gene model was built based on the WS260 annotation. Tag counts for each gene were extracted from STAR aligned BAM files, and differential gene expression between N2 and mutant backgrounds was tested using DESeq2 (Love et al., 2014). A false discovery rate (FDR) < 0.01 and LFC > 0.5849 was used to define genes as upregulated, and FDR < 0.01 and LFC < −1 was used to define genes as downregulated. Table S3 contains the DESeq2 log2 fold change and FDR for each mutant versus wild-type comparison.

Peak calls and annotation to genes

ChIP-seq peaks were called for each factor using YAPC (https://github.com/jurgjn/yapc) (Jänes et al., 2018). Briefly, peak calls were generated through identification of concave regions (regions with negative smoothed second derivative) using the BEADS normalized bigwig tracks. The candidate peaks were tested for statistical significance between replicates using IDR (Li et al., 2011), and only peaks with FDR < 0.001 were kept in our datasets. For three factors (LIN-35, LIN-15B, and EFL-1) we had validated antibodies against the protein; however, to determine LIN-36 binding, we endogenously CRISPR tagged it using GFP. To test that the GFP tag did not disturb the binding of the other factors, we also chromatin immunoprecipitated the other factors in the *lin-36::eGFP* strain. For each factor the Spearman correlation (calculated using DeepTools (Ramírez et al., 2016)) over the peak calls is between 0.76 and 0.98 (Table S2). Therefore, to call wild-type peaks for LIN-35, LIN-15B and EFL-1 we have used all four of our biological replicates, while we have used two for LIN-36. We further redefined these calls by merging overlapping LIN-35, LIN-36 and/or LIN-15B peaks, and then re-scaling merged and factor specific peaks to ± 100bp around their midpoint. The resulting peaks were assigned to genes if they were within the furthest upstream promoter in Jänes et al. (2018) and the end of the gene annotated in Wormbase; if a gene had no promoter annotated in Jänes et al. (2018), peaks were assigned to the gene if they were within the Wormbase start and end positions (Table S2) and the end of the gene (Table S2). Peak overlap with regulatory elements or Dfam2.0 annotated repeats (Hubley et al., 2016) was determined using BEDTools (Quinlan and Hall, 2010).

Identification of direct targets

Direct targets of a given protein were defined as genes upregulated in a mutant condition and that have an associated peak for that protein. The LIN-36-shared and LIN-15B-shared direct targets are direct targets of both LIN-36 and LIN-35, or both LIN-15B and LIN-35, respectively, but not upregulated in *lin-15B* or *lin-36*, respectively (Table S3). Germline expression specificity – defined as the ratio of germline expression (in TPM) to the sum of expression levels from all cell types – of shared targets was determined using L2 single cell RNA-seq data from Cao et al. (2017) (data in Table S3).

GO enrichment analysis

Enrichment for specific gene ontology terms was obtained using the Gene Enrichment Analysis (GEA) tool (Angeles-Albores et al., 2018) available on Wormbase.

Gene body HTZ-1 enrichment

Average gene body HTZ-1 (gbHTZ-1) read coverage was calculated from the region from the most upstream Wormbase TSS +500bp to the most downstream TTS. We identified genes showing a significant loss of HTZ-1 (LFC versus N2 < 0, adjusted p < 0.001) by running DESeq2 on the coding genes in the top 90% of gbHTZ-1 coverage in N2. Genes shorter than 500 bp in length were excluded from the analysis.

H3K9me2 enrichment

Average H3K9me2 signal (BEADS normalized linear coverage) was calculated over LIN-35 + LIN-36 or LIN-35 + LIN-15B ChIP peaks associated to the putative promoter region (i.e., −500 – 0bp upstream of any Wormbase coding transcript) of their respective LIN-36-shared or LIN15B-shared direct targets. Peaks showing a significant loss of H3K9me2 (LFC versus N2 < 0, adjusted p < 0.01) were identified using DESeq2 on LIN-35, LIN-36 and/or LIN-15B peaks overlapping a wild-type H3K9me2 peak (called using MACS; Zhang et al., 2008; with standard settings).

Motif enrichment analysis

DNA motifs enriched at individual factor peaks (with: -objfun de), and at LIN-36-shared and LIN-15B-shared promoter-associated peaks (with: -objfun classic) were detected using MEME (Bailey et al., 2009). Motifs hits in LIN-36-shared and LIN-15B-shared target peaks were annotated using FIMO using a P value cutoff of 0.0001. We included in our final analysis only E2F-a1/a2/b, CDE-CHR-a/b and LONG-a/b motifs as they were found in more than 30% of either set of peaks; we also excluded low-complexity MEME motifs. Overlapping motifs were resolved using the following hierarchy: 1) CDE-CHR; 2) LONG; 3) E2F. UpSet plots were generated through the following Shiny App: https://gehlenborglab.shinyapps.io/upsetr. Similarity to known motifs was evaluated using TOMTOM from the MEME suite.

Identification of differentially bound peaks

DESeq2 was used to identify peaks differentially bound between wild-type and a mutant background by comparing the read counts from the bwa aligned BAM files mapped in wild-type peak regions. Peaks with increased signal in mutants have adjusted *P*-value < 0.001 and LFC > 0. Peaks with decreased signal in mutants have adjusted p < 0.001 and LFC < 0.

Graphical abstract

The Graphical abstract was created with Biorender.com.

QUANTIFICATION AND STATISTICAL ANALYSIS

The significance of the overlap of LIN-15B and LIN-36 direct targets with LIN-35 direct targets was assessed with a hypergeometric test.

Significant differences in a) germline-specificity for LIN-15B shared targets, LIN-36 shared targets and LIN-35-only targets; b) gbHTZ-1 and promoter H3K9me2 signal at LIN-15B and LIN-36 shared targets were determined using a Wilcoxon rank sum test.

Overrepresentation of LIN-15B or LIN-36 shared targets showing a significant loss of gbHTZ-1 or promoter H3K9me2 in different mutant backgrounds was assessed with a hypergeometric test (with BH correction for multiple testing).

Significant differences in LIN-15B and LIN-36 shared targets expression between wild-type and *met-2* strains were determined using a t test.

Significant differences in a) the fraction of LIN-15B and LIN-36 shared targets upregulated in different DREAM components mutants; b) the fraction of LIN-15B and LIN-36 shared targets peaks containing distinct motifs; c) the fraction of LIN-15B and LIN-36 shared targets peaks showing a significant difference in LIN-15B, LIN-15B(ΔTHAP), LIN-36 or LIN-35 ChIP-seq signal in the corresponding factor's mutant compared to wild-type were determined using Fisher's exact tests (with BH correction for multiple testing).

In all figures, significant differences were marked as follows: ns: $p > 0.05$; *:$p < 0.05$, **:$p < 0.01$, ***:$p < 0.001$.

Cell Reports, Volume *37*

Supplemental information

DREAM represses distinct targets

by cooperating with different THAP domain proteins

Csenge Gal, Francesco Nicola Carelli, Alex Appert, Chiara Cerrato, Ni Huang, Yan Dong, Jane Murphy, Andrea Frapporti, and Julie Ahringer

Figure S1

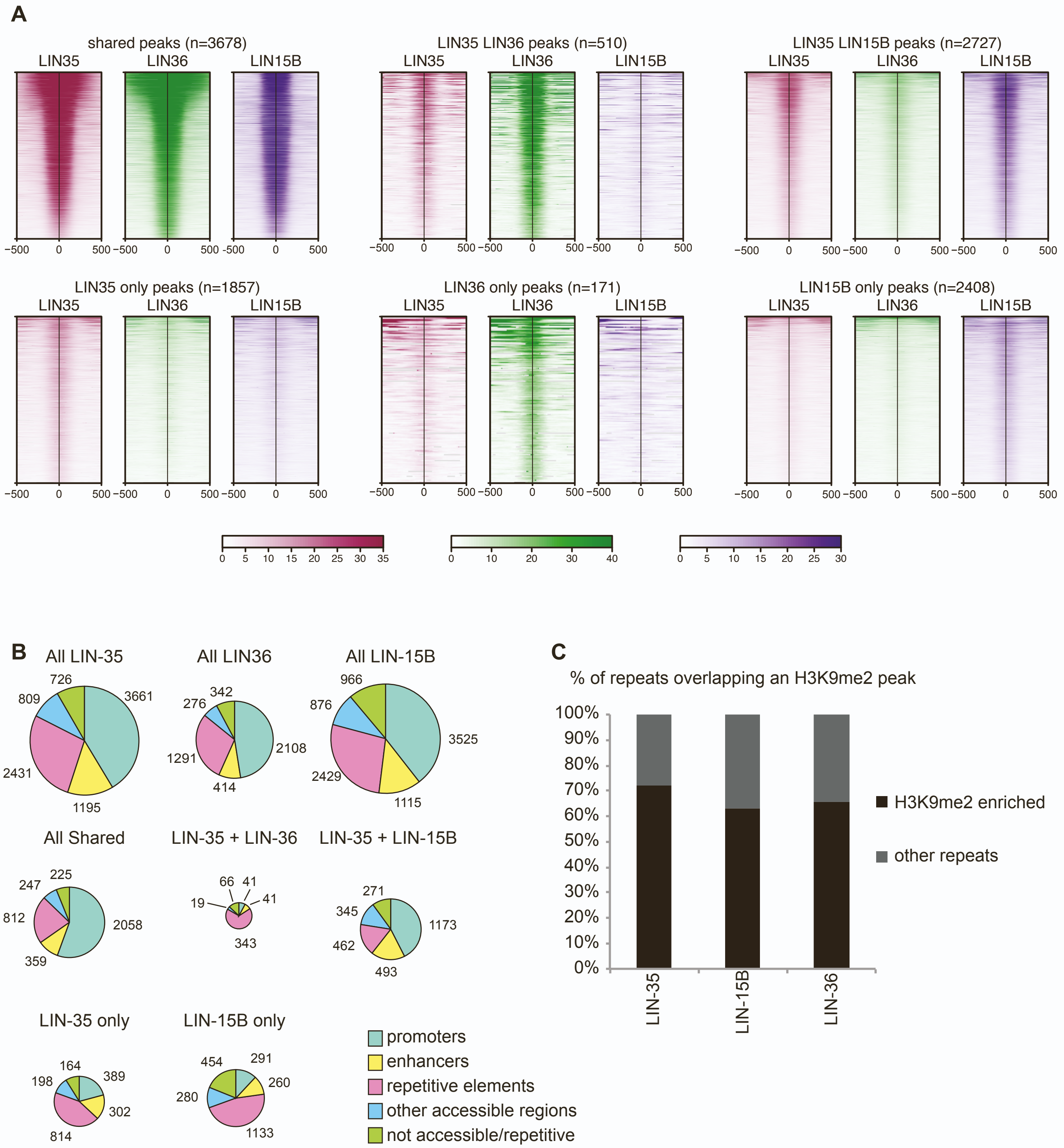
A
shared peaks (n=3678)
LIN35 LIN36 peaks (n=510)
LIN35 LIN15B peaks (n=2727)
LIN35 only peaks (n=1857)
LIN36 only peaks (n=171)
LIN15B only peaks (n=2408)
LIN35
LIN36
LIN15B
-500 0 500
0 5 10 15 20 25 30 35
0 10 20 30 40
0 5 10 15 20 25 30
B
All LIN-35
726 3661 809 2431 1195
All LIN36
342 276 2108 1291 414
All LIN-15B
966 876 3525 2429 1115
All Shared
225 247 812 2058 359
LIN-35 + LIN-36
66 41 19 41 343
LIN-35 + LIN-15B
271 345 1173 462 493
LIN-35 only
164 198 389 302 814
LIN-15B only
454 291 260 280 1133
promoters
enhancers
repetitive elements
other accessible regions
not accessible/repetitive
C
% of repeats overlapping an H3K9me2 peak
100%
90%
80%
70%
60%
50%
40%
30%
20%
10%
0%
LIN-35
LIN-15B
LIN-36
H3K9me2 enriched
other repeats

Figure S1. LIN-35, LIN-36 and LIN-15B co-localize extensively on chromatin, related to Figure 1. (A) Heatmaps of BEADS normalized ChIP-seq coverage centred over the indicated regions. Tracks are from combined replicates. We note that signal at single factor sites is generally weak and therefore confidence that other factors are not present is not high. (B) Assignments of peaks to features in the genome. Peaks were first overlapped with regulatory elements identified in Janes *et al*. 2018, then with repetitive elements from Dfam2.0. (C) Fraction of LIN-35, LIN-15B and LIN-36-bound repeats (from Dfam 2.0) overlapping H3K9me2 ChIP-seq peaks.

Figure S2

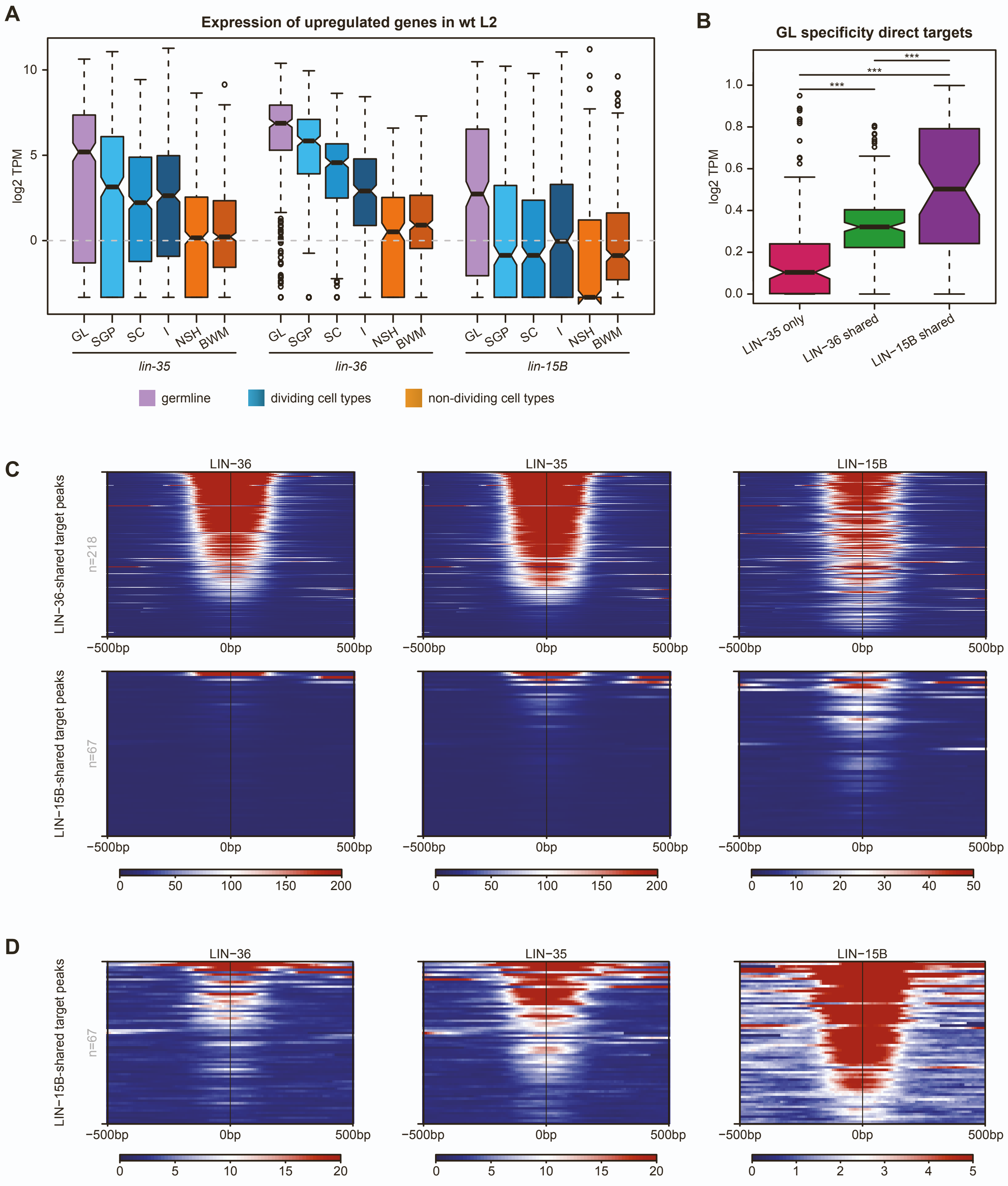

A
Expression of upregulated genes in wt L2
log2 TPM
GL
SGP
SC
I
NSH
BWM
lin-35
lin-36
lin-15B
germline
dividing cell types
non-dividing cell types
B
GL specificity direct targets
log2 TPM
***
LIN-35 only
LIN-36 shared
LIN-15B shared
C
LIN-36
LIN-35
LIN-15B
LIN-36-shared target peaks
n=218
LIN-15B-shared target peaks
n=67
−500bp
0bp
500bp
D
LIN-36
LIN-35
LIN-15B
LIN-15B-shared target peaks
n=67

Figure S2. Expression and binding profile of LIN-35, LIN-36 and LIN-15B targets, related to Figure 1. (A) Expression level (measured as log2 TPM) of genes upregulated in *lin-35*, *lin-36(we36)*, and *lin-15B* in the germline and in different types of dividing (SGP: somatic gonad precursors, SC: seam cells, I: intestine) and non-dividing (NSH: non-seam hypodermis, BWM: body wall muscle) cell types. The dashed grey line indicates a TPM value of 1. Expression difference between germline and any other tissue was significant for LIN-35, LIN-36 and LIN-15B targets (Benjamini-Hochberg adjusted Mann-Whitney test $P<10^{-3}$) (B) Germline expression specificity (calculated as expression in germline / sum of expression in any cell type) of LIN-35-specific, LIN-36-shared and LIN-15B-shared direct targets. (C) and (D) Heatmaps of BEADS normalized ChIP-seq coverage centred over the LIN-35+LIN-36 and LIN35+LIN-15B peaks associated to the promoters of LIN-36-shared (C) and LIN-15B-shared (C and D, in different scales) direct targets. Significant differences (Wilcoxon rank sum test): ***: $P<0.001$. Expression data for panels (A, B) are from Cao et al, 2017.

Figure S3

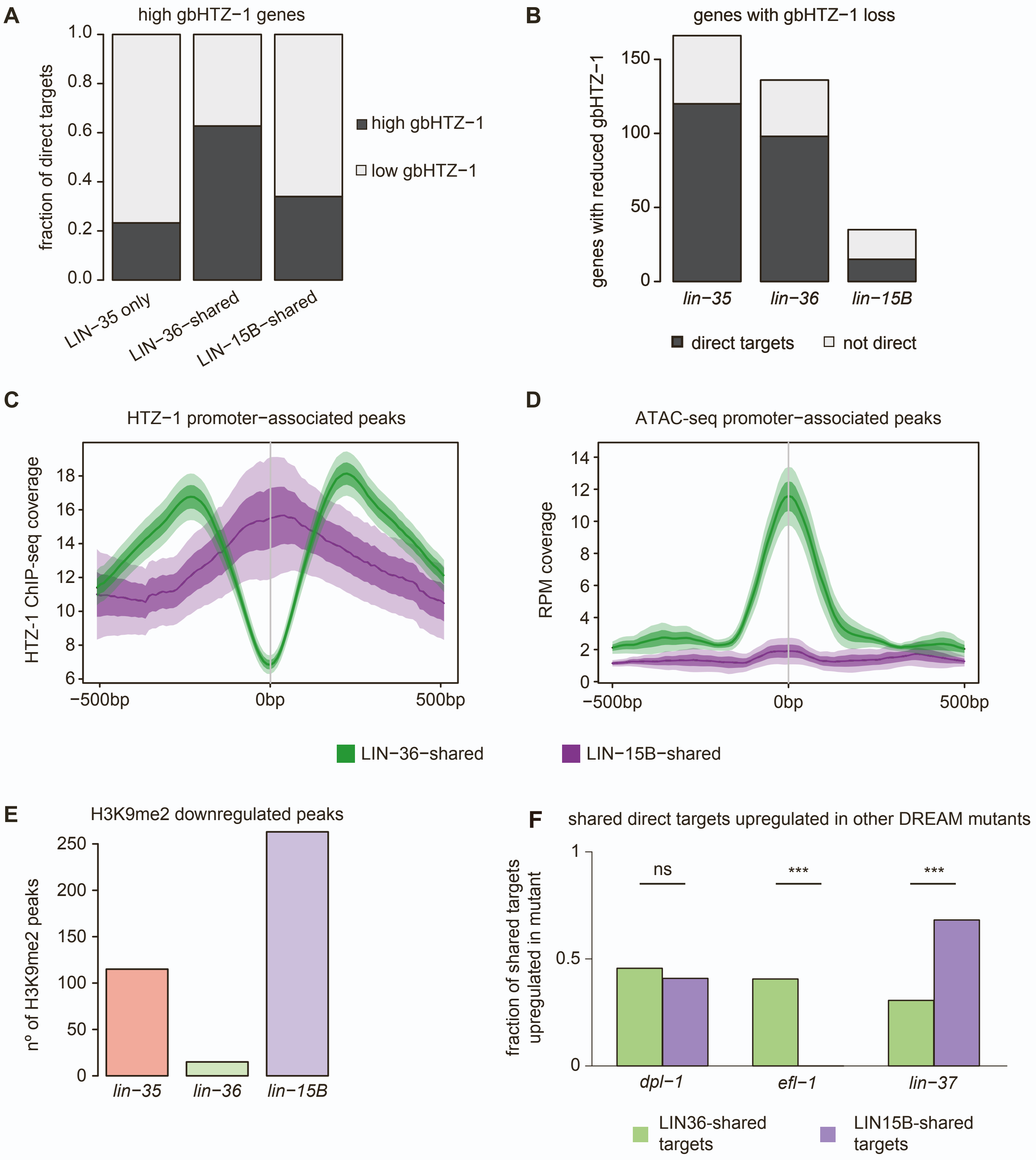
A
high gbHTZ−1 genes
fraction of direct targets
1.0
0.8
0.6
0.4
0.2
0.0
LIN−35 only
LIN−36−shared
LIN−15B−shared
high gbHTZ−1
low gbHTZ−1
B
genes with gbHTZ−1 loss
genes with reduced gbHTZ−1
150
100
50
0
lin−35
lin−36
lin−15B
direct targets
not direct
C
HTZ−1 promoter−associated peaks
HTZ-1 ChIP-seq coverage
18
16
14
12
10
8
6
−500bp
0bp
500bp
D
ATAC-seq promoter−associated peaks
RPM coverage
14
12
10
8
6
4
2
0
−500bp
0bp
500bp
LIN−36−shared
LIN−15B−shared
E
H3K9me2 downregulated peaks
nº of H3K9me2 peaks
250
200
150
100
50
0
lin−35
lin−36
lin−15B
F
shared direct targets upregulated in other DREAM mutants
fraction of shared targets upregulated in mutant
1
0.5
0
ns
***
***
dpl−1
efl−1
lin−37
LIN36-shared targets
LIN15B-shared targets

Figure S3. Different silencing mechanisms of LIN-36-shared targets and LIN-15B-shared targets, related to Figure 2. (A) fraction of LIN-35-specific, LIN-36-shared and LIN-15B-shared direct targets with high (top 10% across all coding genes, dark grey) or low (bottom 90%, light grey) levels of gbHTZ-1. (B) Number of coding genes showing a significant reduction in gbHTZ-1 levels in the respective mutants. Dark grey bars indicate direct targets. (C) Signal plot of BEADS normalised HTZ-1 ChIP-seq coverage calculated over the LIN-35+LIN-36 and LIN35+LIN-15B peaks associated to the promoters of LIN-36-shared (green) and LIN-15B-shared (purple) direct targets. (D) Signal plot of ATAC-seq signal (in RPM coverage) from L1-staged larvae over the LIN-35+LIN-36 and LIN35+LIN-15B peaks associated to the promoters of LIN-36-shared (green) and LIN-15B-shared (purple) direct targets. ATAC-seq data from Janes et al., 2018. (E) Number of LIN-35, LIN-36 and/or LIN-15B peaks showing a significant reduction in H3K9me2 levels in mutants. (F) Fraction of LIN-36-shared (green) and LIN-15B-shared (purple) direct targets showing upregulated expression in *dpl-1*, *efl-1* and *lin-37* mutants. Significant differences (LIN-36-shared vs LIN-15B-shared fraction, Fisher's exact test with Benjamini-Hochberg correction): ***: $P<0.001$; ns: $P>0.05$.

Figure S4

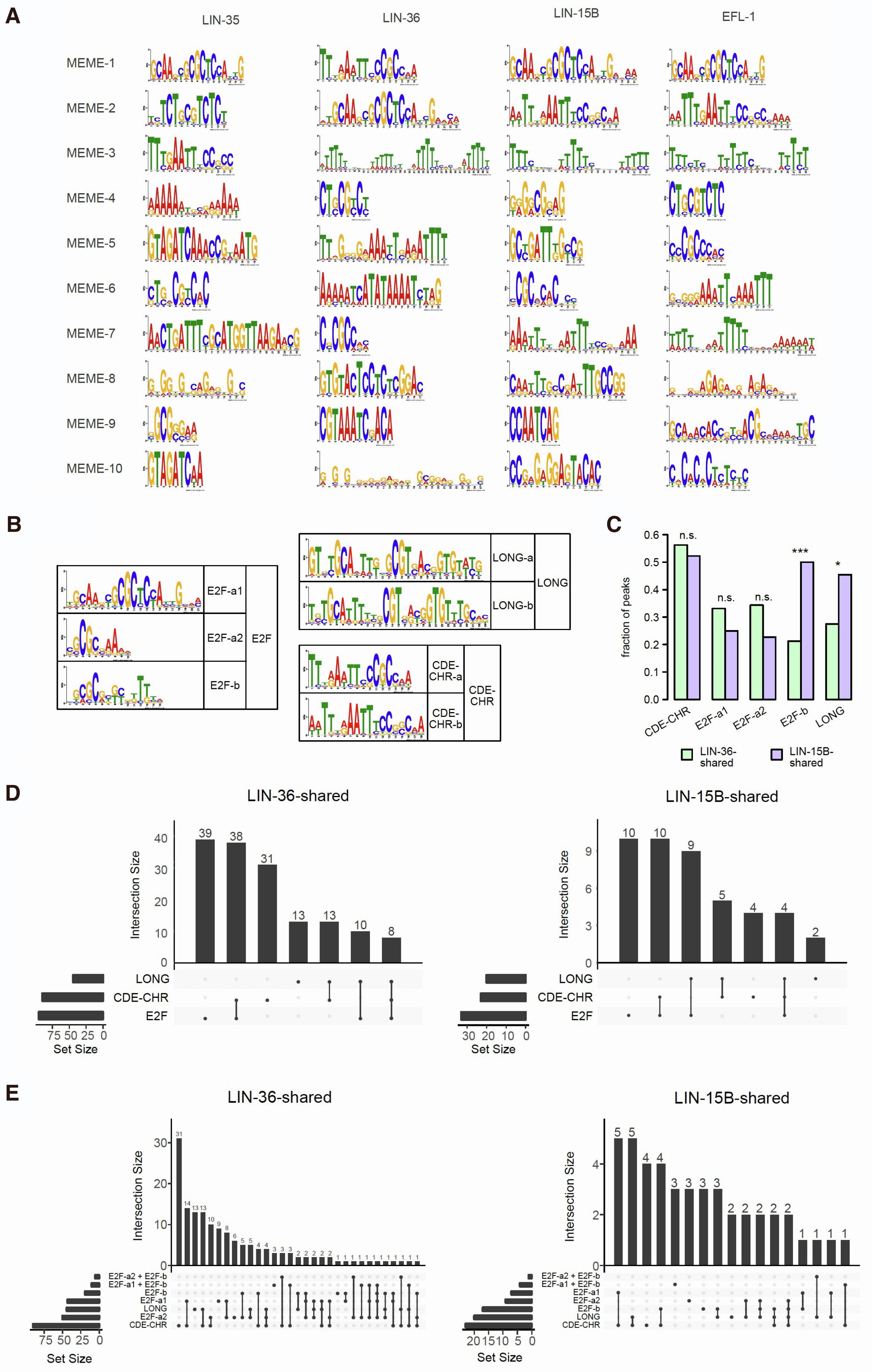
A
LIN-35
LIN-36
LIN-15B
EFL-1
MEME-1
MEME-2
MEME-3
MEME-4
MEME-5
MEME-6
MEME-7
MEME-8
MEME-9
MEME-10
B
E2F-a1
E2F-a2
E2F-b
E2F
LONG-a
LONG-b
LONG
CDE-CHR-a
CDE-CHR-b
CDE-CHR
C
fraction of peaks
n.s.
n.s.
n.s.
***
*
CDE-CHR
E2F-a1
E2F-a2
E2F-b
LONG
LIN-36-shared
LIN-15B-shared
D
LIN-36-shared
Intersection Size
39
38
31
13
13
10
8
LONG
CDE-CHR
E2F
Set Size
LIN-15B-shared
10
10
9
5
4
4
2
E
LIN-36-shared
E2F-a2 + E2F-b
E2F-a1 + E2F-b
E2F-b
E2F-a1
LONG
E2F-a2
CDE-CHR
LIN-15B-shared

Figure S4, Motifs enriched at LIN-35, LIN-36, LIN-15B and EFL-1 peaks, related to Figure 1. (A) Logos of motifs enriched in full sets of LIN-35, LIN-36, LIN-15B and EFL-1 peaks. (B) E2F, LONG and CDE-CHR motif variants enriched at LIN-36-shared and LIN-15B-shared target promoters. (C) Fraction of LIN-36-shared and LIN-15B-shared promoter-associated peaks containing each motif. For CDE-CHR and LONG motifs, we included sites corresponding to both variant. Significant differences (Fisher's exact test with Benjamini-Hochberg correction): ***: $P < 0.001$; *: $P < 0.05$; ns: $P > 0.05$. (D-E) UpSet plots showing the co-occurrence of each motif class (D) or of individual E2F variants with LONG and CDE-CHR motifs (E) in LIN-36-shared and LIN-15B-shared target peaks.

Figure S5

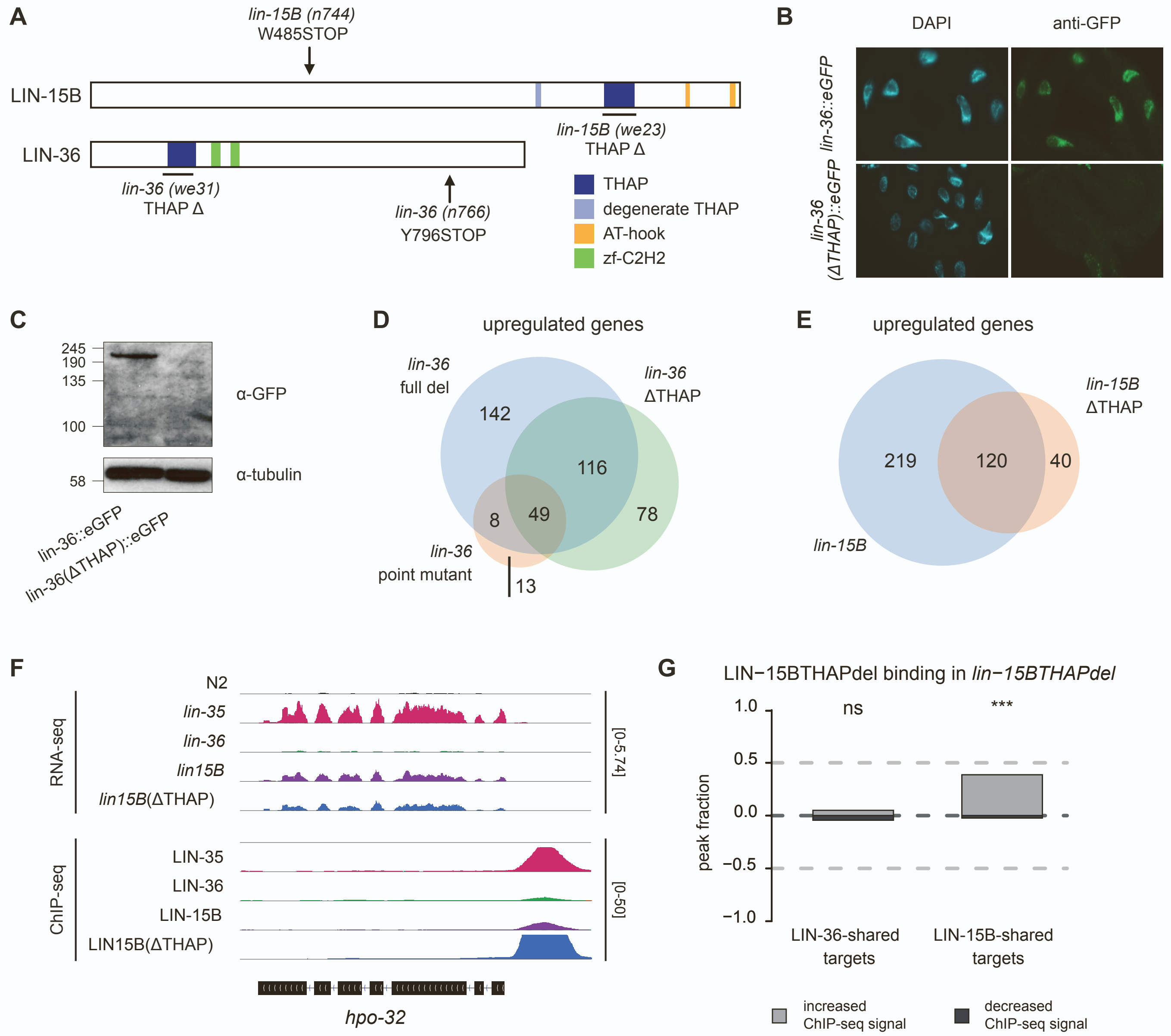
A
lin-15B (n744)
W485STOP
LIN-15B
lin-15B (we23)
THAP Δ
LIN-36
lin-36 (we31)
THAP Δ
lin-36 (n766)
Y796STOP
THAP
degenerate THAP
AT-hook
zf-C2H2
B
DAPI
anti-GFP
lin-36::eGFP
lin-36 (ΔTHAP)::eGFP
C
245
190
135
100
58
α-GFP
α-tubulin
lin-36::eGFP
lin-36(ΔTHAP)::eGFP
D
upregulated genes
lin-36
full del
lin-36
ΔTHAP
142
116
8
49
78
lin-36
point mutant
13
E
upregulated genes
lin-15B
ΔTHAP
219
120
40
lin-15B
F
RNA-seq
N2
lin-35
lin-36
lin15B
lin15B(ΔTHAP)
[0-5.74]
ChIP-seq
LIN-35
LIN-36
LIN-15B
LIN15B(ΔTHAP)
[0-50]
hpo-32
G
LIN−15BTHAPdel binding in lin−15BTHAPdel
1.0
0.5
0.0
−0.5
−1.0
peak fraction
ns
***
LIN-36-shared
targets
LIN-15B-shared
targets
increased
ChIP-seq signal
decreased
ChIP-seq signal

Figure S5. Effects of THAP domain deletion in LIN-36 and LIN-15B, related to Figure 3. (A) Diagram of the LIN-15B and LIN-36 proteins, illustrating their predicted DNA binding domains and the deletions generated in this study. Arrows indicate the positions of the premature stop codons in the corresponding alleles. The lin-15B(ΔTHAP) allele did not remove the highly degenerate, upstream THAP-like domain. (B) Anti-GFP immunofluorescence of the LIN-36::eGFP fusion protein in the *lin-36::eGFP* and *lin-36(ΔTHAP)::eGFP* strains. No GFP signal was detected in *lin-36(ΔTHAP)::eGFP* worms. C) Western blot with anti-GFP antibody was used to detect the LIN-36::eGFP and LIN-36(ΔTHAP)::eGFP proteins; the band corresponding to the LIN-36(ΔTHAP)::eGFP protein was not detected. (D) and (E) Overlap between genes upregulated in (D) *lin-36* or (E) *lin-15B* mutant strains. (F) IGV view of a representative LIN-15B-shared direct target. Factor-specific ChIP-seq enrichment shown as BEADS-normalized coverage. RNA sequencing data depict read-depth normalized coverage from combined replicates. (G) Fraction of LIN-36-shared (left) and LIN-15B-shared (right) promoter-associated LIN15B(ΔTHAP) peaks showing a significant difference in occupancy in *lin-15B(ΔTHAP)* mutants. Significant differences (up- vs downregulated fraction, Fisher's exact test with Benjamini-Hochberg correction): ***: $P < 0.001$; ns: $P > 0.05$.
